# Global dietary estimates for conducting health, environmental and economic impact assessments

**DOI:** 10.1038/s43016-026-01388-z

**Published:** 2026-07-03

**Authors:** Marco Springmann

**Affiliations:** 1 Institute for Global Health, University College London, London, UK; 2 Environmental Change Institute, University of Oxford, Oxford, UK

**Keywords:** Society, Geography

## Abstract

Dietary intake has important implications for health, the environment and the economy. However, current global estimates of food intake are uncertain and incomplete. Here we develop a Global Dietary Database for Impact Assessments (GDD-IA) that combines data sources on regional food availability and food waste estimates, socio-demographic variation in intake from dietary surveys and energy intake estimates based on measurements of body weight, height and physical activity. The GDD-IA covers food intake of 43 food groups by country, age, sex and urban/rural residence across five-year periods between 1990 and 2020. It is designed for dietary impact assessments that require coverage of complete diets, absolute food intake levels that minimize risk of over or underestimation and regional comparability with granular socio-demographic detail. We illustrate the advantages of using the GDD-IA over unadjusted dietary-survey and food availability estimates by assessing the health burden, environmental resource use and food costs related to diets.

## Abstract (web summary)

The Global Dietary Database for Impact Assessments provides information on what people eat across countries and demographic groups by integrating data on food availability, consumption and waste, as well as energy needs. It supports analyses of the impacts of diets on health, environmental and economic outcomes.

## Main

The impacts our diets have on our health and the health of the environment are two of the great challenges of our times^1,2^. Imbalanced diets pose one of the greatest health burdens and are estimated to be responsible for one in five deaths globally^3,4^. Eating too many calories and the associated levels of overweight and obesity have also been increasing and are now affecting over 2 billion people, whereas eating too few calories remains a persistent problem for about 800 million people^5,6^. The food systems producing those diets are major contributors to biodiversity loss^7^, the pollution of terrestrial and aquatic systems^8–10^ and climate change^11^, including the threats to livelihoods and public health associated with it^12,13^.

A key input for assessing the impacts our diets have on health and the environment are estimates of food intake. Despite their importance, existing estimates and global databases of food intake have substantial shortcomings^14,15^. Some of the most commonly used data in impact assessments are estimates of how much food is available in a country. The data are provided and regularly updated by the Food and Agriculture Organization of the United Nations (FAO)^16,17^. However, food availability estimates have been found to overestimate actual intake if not carefully adjusted for food waste^18,19^, and estimates of food waste are rarely updated^20^. Because food availability estimates are based on nationally reported statistics, they also do not include subsistence production of food for own consumption, which can be particularly important in low-income regions with a large proportion of small-holder farmers^21,22^. Lastly, they lack data on the socio-demographic characteristics of food intake, which makes them not well suited for guiding public health interventions^21,23^.

Another source of data are dietary surveys, including 24-hour recalls and food-frequency questionnaires. They provide a rich source of information, including on socio-demographic characteristics, and are considered the gold standard in public health research^15^. However, they are prone to misreporting, for example, due to imprecision of communicating and reporting serving sizes^24^, difficulties in the recall of intake^15^ and tendencies in responders to report what is perceived as socially desirable^25,26^. As a result, dietary surveys are known to substantially underestimate total calorie intake^27–31^. Datasets such as the Global Dietary Database (GDD) have standardized and harmonized individual-level dietary surveys, but they normalized food intake to the same total energy intake in every country and most socio-demographic groups and therefore do not capture actual food intake^32,33^. In addition, the GDD focusses on a selection of food groups and therefore does not capture complete diets, which makes the estimates unsuitable for assessments for which overall intake matters, including food security, affordability, nutritional adequacy and environmental impacts.

Here we develop a new global dietary database that is particularly designed for use in impact assessments that require coverage of complete diets, absolute intake levels that minimize risk of over or underestimation and granular socio-demographic detail. Examples of such impact assessments include analyses of nutritional adequacy, dietary risk assessments, environmental analyses of diets^34,35^, estimates of the affordability of diets^36^ and assessments of the proportion of undernourishment^37,38^. For constructing the Global Dietary Database for Impact Assessments (GDD-IA), we combined the key strengths of existing data and used newly estimated data to address existing shortcomings. To derive the composition and overall scale of dietary intake, we used estimates of food available in a country provided by the FAO in their food balance sheets (FBSs)^17^ and first subtracted estimates of food waste at retail and household levels^20^ and then normalized intake to estimates of total energy intake that are based on anthropometric measures of body weights, heights and physical activity^39^. This normalization procedure ensured that our estimates are biophysically plausible and represent absolute levels of intake.

In addition, we used estimates from dietary surveys harmonized by the GDD to derive the degree of variation in intake across socio-demographic groups and for estimating the intake of processed foods. For the former, we mapped the variation in intake across sex, age groups and urban/rural residence inferred from the GDD dietary surveys^32^ to the food group detail of the FBS-based national estimates and normalized them before and after the mapping to the estimated energy intake in those population groups^39^. As before, the normalization ensures the estimates are biophysically plausible, whereas the mapping downscales the GDD survey estimates from general food groups (for example, grains) to specific commodities (for example, wheat). For estimating intake of processed foods, we used the normalized survey estimates to derive processing ratios of red meat into processed meat and of grains into refined grains and applied them to our estimated intake. For processed dairy, we used estimates of FAO’s supply utilization accounts to split milk equivalents into cheese and yogurt.

The estimates are available in Supplementary Datafiles^40^ and will also be made available via an interactive data explorer^41^. Therein, we also compare our estimates of food intake to estimates based on dietary surveys, and we developed additional proxies of intake that can be used in structured sensitivity analyses, including estimates that are more directly based on survey estimates, either for general food groups or for foods relevant to subsistence farming that might not be well captured by food availability statistics. Below, we provide an overview of the intake estimates, compare them to estimates from dietary surveys and illustrate how they improve health, environmental and economic impact assessments related to diets.

## Results

**Fig. 1:**
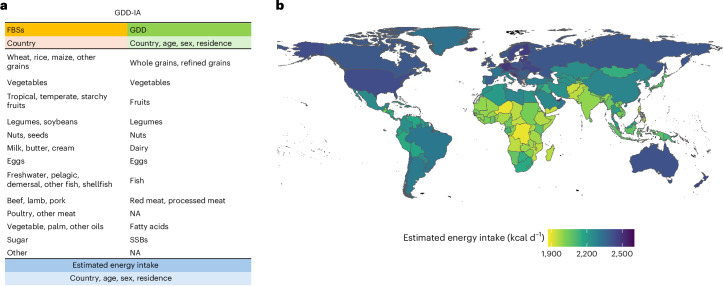
Socio-demographic and food group coverage of the data sources that are combined in the GDD-IA. **a**,**b**, Dietary composition at the country level was derived from food availability statistics contained in FBSs adjusted for food waste (**a**) and normalized to estimates of total energy intake based on anthropometric measurements (**b**). Variation in intake across socio-demographic groups was derived from the Global Dietary Database for general food groups (**a**) and again normalized to estimated energy intake for each population group (**b**). The colour shading in **a** indicates data sources (dark shading) and socio-demographic detail (light shading). SSBs, sugar-sweetened beverages; NA, food groups that are not available in the data source.

The data sources used to construct the GDD-IA differed in their coverage of food groups and socio-demographic details (Fig. 1). The data on food available for human consumption from FAO’s FBSs covered 92 food commodities and five beverages by country for the years 1990 to 2020 (with estimates reaching back to 1961). The data on reported intake from dietary surveys included in the GDD covered 14 food groups and seven beverages by country, age, sex, education and urban/rural residence for the years 1990 to 2018. The data on estimated energy requirements covered total energy intake by country, age, sex and urban/rural residence for the years 1990 to 2020. We combined and harmonized the data (Methods) to cover food intake (in grams, kilocalories and servings per person and day) for 43 food groups by country, age, sex and urban/rural residence for every five years between 1990 and 2020 (Table 1).

**Table 1:** Food intake in 2020 by food category, food group and unit of intake

Food category	Food group	servings per day			g d^−1^			kcal d^−1^		
		mean	low	high	mean	low	high	mean	low	high
staples	wheat	2.6	2.5	2.7	116	112	120	344	332	358
	rice	4.0	3.9	4.1	180	173	186	441	426	457
	maize	0.7	0.7	0.8	33	31	34	100	97	105
	other grains	0.4	0.4	0.5	20	19	21	58	56	61
	roots	1.4	1.3	1.4	136	131	142	116	111	121
fruits and vegetables	vegetables	2.4	2.3	2.4	236	228	244	59	57	62
	tropical fruits	0.4	0.4	0.4	41	40	43	20	20	21
	temperate fruits	0.7	0.7	0.7	68	66	71	32	31	33
	starchy fruits	0.3	0.3	0.3	30	29	32	21	20	22
legumes	legumes	0.4	0.4	0.4	19	18	20	65	63	68
	soybeans	0.1	0.1	0.1	4	4	4	14	13	14
nuts and seeds	nuts	0.4	0.4	0.5	11	11	12	39	38	41
	seeds	0.0	0.0	0.0	1	1	1	5	5	5
oils	vegetable oils	1.5	1.4	1.5	22	21	23	210	203	218
	palm and coconut oils	0.5	0.5	0.5	7	7	7	68	65	70
	fish oil	0.0	0.0	0.0	0	0	0	0	0	0
sugar	sugar	5.8	5.6	6.1	47	45	49	149	144	155
dairy and fats	milk equivalents	0.7	0.7	0.7	172	165	179	130	126	136
	butter	0.8	0.7	0.8	4	4	4	30	29	31
	cream	0.1	0.1	0.1	1	1	1	2	2	2
	animal fat	0.1	0.1	0.1	2	2	2	24	23	25
eggs	eggs	0.5	0.5	0.5	25	24	26	36	35	37
red meat	beef	0.2	0.2	0.2	17	16	18	26	25	27
	lamb	0.0	0.0	0.0	4	4	4	7	7	8
	pork	0.2	0.2	0.3	24	24	25	68	66	70
other meat	poultry	0.3	0.3	0.3	28	27	29	39	38	41
	other meat	0.0	0.0	0.0	1	1	1	1	1	2
fish	freshwater fish	0.1	0.1	0.1	9	9	10	6	6	7
	pelagic fish	0.0	0.0	0.0	4	4	4	4	3	4
	demersal fish	0.0	0.0	0.0	3	3	3	2	2	2
	other fish	0.1	0.1	0.1	5	5	5	2	2	2
	shellfish	0.1	0.1	0.1	6	6	6	2	2	2
other	stimulants	0.7	0.6	0.7	3	3	3	4	4	4
	spices	2.1	2.0	2.2	3	3	3	8	8	9
	alcohol	0.4	0.4	0.4	53	51	55	36	35	37
	other	0.4	0.3	0.4	2	2	2	1	1	1
processed foods	whole grains	1.1	1.1	1.1	49	47	51	135	130	141
	processed grains	6.6	6.4	6.9	299	288	311	809	780	841
	red meat	0.3	0.3	0.4	34	33	36	80	78	83
	processed meat	0.2	0.2	0.2	11	10	11	21	20	21
	yogurt	0.0	0.0	0.0	3	3	3	2	2	2
	cheese	0.5	0.4	0.5	8	8	8	26	25	27
	milk	0.5	0.5	0.5	130	125	135	102	98	106
total	energy							2,173	2,095	2,259

The estimates represent population-weighted averages across all countries and age groups. Food intake is expressed per person and day in servings (servings per day), grams (g d^−1^) and kilocalories (kcal d^−1^). The category of processed foods differentiates some previously listed primary foods by their degree of processing. Serving sizes are listed in Supplementary Table 4. Estimates of food intake by country and socio-demographic group are available in the Supplementary Datafiles^40^.

### Estimated food intake across scales

**Fig. 2:**
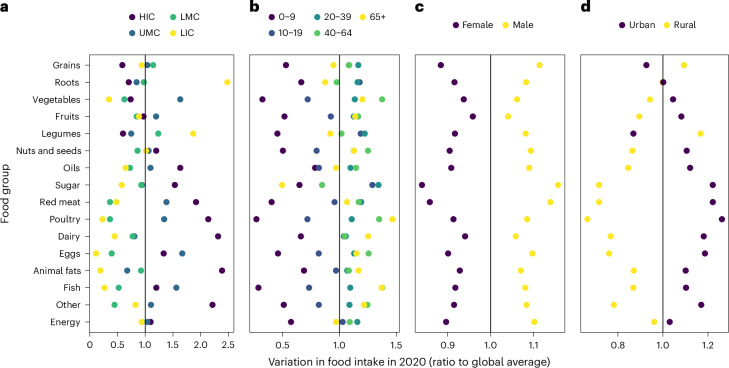
Variation in food intake in 2020 by food group and income region, age group, sex and residence. The variation is expressed in comparison to the global average (Table 1). **a**, The income regions include high-income countries (HIC), upper middle-income countries (UMC), lower middle-income countries (LMC) and low-income countries (LIC) as defined by the World Bank. **b**, Age groups include children (aged 0–9 years), adolescents (aged 10−19 years), young adults (aged 20−39 years), middle-aged adults (aged 40−64 years) and senior adults (aged 65+ years). **c**, Sexes include females and males. **d**, Residences include urban and rural.

In our dataset, dietary intake differs across socio-demographic groups and regions. In 2020, diets on average (Table 1) contained about 7.5–8.0 servings per person and day (servings per day; Supplementary Table 4) of grains (with the range referring to the uncertainty of our estimates); 5.5–6.0 servings per day of sugar; 2.0–2.5 servings each of vegetables and vegetable oils; 1.0–1.5 servings per day each of roots, fruits and animal fats; 0.5–0.7 servings per day each of dairy, nuts and seeds, eggs and red meat and 0.25–0.30 servings per day each of poultry and fish. Across socio-demographic groups, differences in food intake were in line with changes in energy intake for most foods (Fig. 2). Compared to the global population average (2,160 kilocalories per person per day, kcal d^−1^), energy intake was 40% lower in children, 5% higher in adolescents, 15% higher in young adults, 10% higher in middle-aged adults and 5% lower in older adults (Supplementary Table 5). Across sexes, it was 20% lower in women compared to men (mostly reflecting physiological differences), and across residence, it was 5% lower in rural residences compared to urban residences (reflecting income-dependent dietary transitions). The variations differed by region but with similar trends across socio-demographic groups within each region.

Across regions, diets and energy intake differed substantially (Fig. 2). Compared to the global population average, total energy intake ranged from 7% lower than average in low-income countries to 10% greater in high-income countries. Compared to average diets, diets in high-income countries contained about 40% less grains and legumes and 25–30% less roots and vegetables but 90–130% more dairy, animal fats, poultry and red meat; 55–65% more vegetable oils and sugar and 20–30% more nuts and seeds, fish and other foods. Diets in upper middle-income countries contained 55–70% more eggs, vegetables and fish; 30–40% more red meat and poultry and 20% more fruits but also 50% less animal fats and 15–25% less legumes, dairy and roots. Diets in lower middle-income countries, contained 15–25% more legumes, animal fats and grains but also 60–65% less poultry, red meat and eggs, 40–50% less fish and vegetables and 15–30% less vegetable oils, dairy and fruits. Diets in low-income countries contained 150% more roots and 90% more legumes but 75–90% less eggs, animal fats and fish; 50–55% less dairy and red meat and 35–40% less sugar and vegetable oils.

**Fig. 3:**
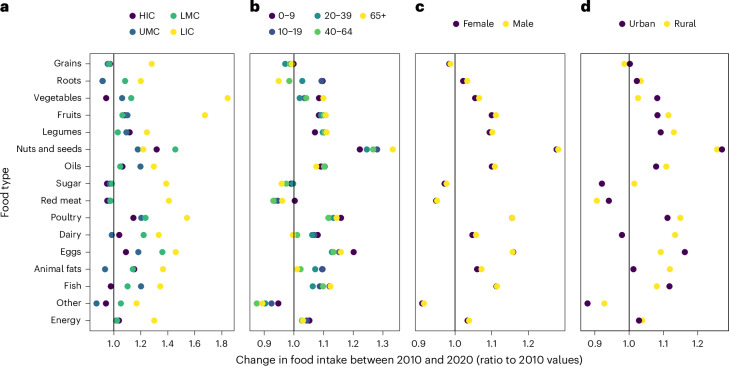
Change in food intake between 2010 and 2020 by food group and income region, age group, sex and residence. Changes are expressed in comparison to values in 2010. **a**, The income regions include high-income countries (HIC), upper middle-income countries (UMC), lower middle-income countries (LMC) and low-income countries (LIC) as defined by the World Bank. **b**, The age groups include children (aged 0−9 years), adolescents (aged 10−19 years), young adults (aged 20−39 years), middle-aged adults (aged 40−64 years) and senior adults (aged 65+ years). **c**, The sexes include females and males. **d**, The residences include urban and rural.

Dietary intake changed over time (Supplementary Fig. 1). For example, in the decade between 2010 and 2020, global food intake increased for most food groups (Fig. 3). This included increased intake of nuts and seeds (+28% on average), poultry and eggs (+16% each), fish and fruits (+11% each), legumes and oils (+10% each) and animal fats, vegetables and dairy (5–7%), whereas intake of sugar and red meat decreased slightly (−3–5%). High-income and middle-income countries showed similar trends but with relatively greater changes in middle-income countries. In contrast, food intake in low-income countries increased for all food groups and in greater proportions, ranging from increases of 20% for roots to 40–55% for poultry and red meat and to 70–85% for vegetables and fruits. Total energy intake increased by 4% globally, ranging from 2% in middle-income countries to 4% in high-income countries and to 30% in low-income countries. The increases in energy intake were relatively greater in children and adolescents (4–5%) than in adults (3%) in most regions, slightly greater in men (4%) compared to women (3.5%) and in rural residences (4%) compared to urban ones (3%).

### Comparison to estimated intake from dietary surveys

**Fig. 4:**
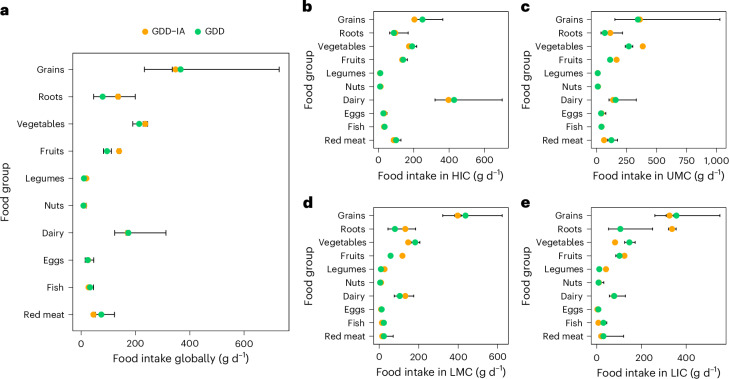
Comparison of food intake between the GDD-IA and the GDD by region and food group. **a**–**e**, The regions include a global average (**a**) and high-income countries (**b**; HIC), upper middle-income countries (**c**; UMC), lower middle-income countries (**d**; LMC) and low-income countries (**e**; LIC). The error bars denote the low and high values of the estimates’ uncertainty ranges.

At the global level, the uncertainty intervals of the new GDD-IA estimates of food intake overlapped with the uncertainty intervals of dietary-survey estimates from the GDD for most food groups (seven out of ten) when normalized to the same energy intake (Methods), except for fruits, legumes and red meat (Fig. 4). However, the uncertainty intervals of the survey-based estimates were large and incorporated substantial variation in mean values, including for red meat (+65% in the survey-based estimates compared to the main ones), fish (+20%) and for legumes (−45%), roots (−40%), fruits (−30%) and nuts (−25%). The large range of uncertainty intervals resulted in a range of total energy intake of 1,620 to 3,615 kcal d^−1^ when gap-filled with estimates from our main GDD-IA proxy for foods not represented in the GDD dataset of survey estimates (that is, of foods that make up complete diets but are not represented in the GDD such as poultry and oils (Methods and Supplementary Table 11)).

The direction of variation differed across regions (Fig. 4). Although the uncertainty ranges overlapped for all food groups in high-income countries, the mean values of the survey-based estimates from the GDD were substantially greater for grains (+20%, +45 g d^−1^) and substantially smaller for nuts (−25%, −3 g d^−1^). In upper middle-income countries, the survey-based estimates were substantially lower for roots (−40%, −45 g d^−1^), statistically (that is, without overlapping uncertainty intervals) lower for vegetables (−30%, −115 g d^−1^) and fruits (−30%, −55 g d^−1^) and statistically greater for red meat (+95%, +60 g d^−1^), which exceeded food availability statistics from the FBS without waste subtracted. In lower middle-income countries, the survey-based estimates were statistically lower for legumes (−65%, −20 g d^−1^), fruits (−50%, −60 g d^−1^), statistically greater for vegetables (+25%, +35 g d^−1^) and fish (+70%, +10 g d^−1^) and showed substantial difference but within uncertainty ranges for red meat (+55%, +10 g d^−1^). In low-income countries, the survey-based estimates were statistically lower for legumes (−75%, −30 g d^−1^) and roots (−70%, −230 g d^−1^) and statistically greater for fish (+320%, +25 g d^−1^), eggs (+155%, +5 g d^−1^) and vegetables (+80%, +65 g d^−1^).

### Example applications in dietary impact assessments

**Fig. 5:**
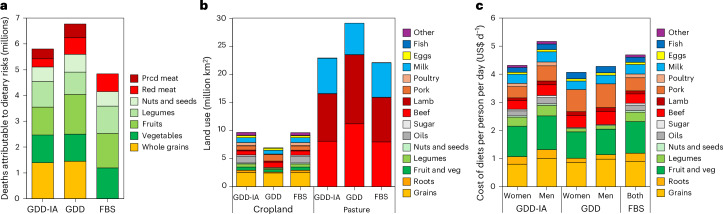
Comparison of estimates of intake in dietary impact assessments for the year 2015. **a**–**c**, Comparisons of the diet-related disease burden (**a**), environmental resource use (**b**) and the cost of diets (**c**). The estimates of intake include the GDD-IA, estimates from dietary surveys contained in the GDD and waste-adjusted food availability data from FAO’s FBSs. The assessment of health impacts (**a**) displays the estimated number of deaths attributable to dietary risks globally by dietary data source and risk factor. The risk factors include low intake of whole grains, vegetables, fruits, legumes, nuts and high intake of red meat and processed meat. The assessment of environmental resource use (**b**) displays the estimated use of cropland and pasture related to diets by dietary data source and food group. The cost assessment (**c**) displays the cost of diets per person per day by dietary data source, sex and food group. Uncertainty intervals are reported in the Supplementary Information (Supplementary Tables 13–15). Prcd meat, processed meat; veg, vegetables.

We conducted a series of impact assessments (Methods) to illustrate the relative differences between the new database (GDD-IA), the global database of dietary surveys (GDD) and waste-adjusted food availability data (FBS). In a health assessment based on a comparative risk assessment of dietary risks (Fig. 5a and Supplementary Table 13), we found that using the GDD-IA resulted in a lower estimate of deaths attributable to dietary risks as when using the GDD data (−750,000 globally; −12%), but in a higher number as when using FBS-based data (+780,000; +17%). In the former, the relative differences were particularly large for the Middle East (−55,000; −19%) and East Asia (−290,000; −15%) and mostly due to the lower estimated intake of fruits and vegetables and greater estimated intake of red meat in the survey-based GDD data, both of which resulted in a greater attributable burden. In the latter, the relative differences were particularly large for North America (+80,000; +29%) and Latin America (+70,000; +25%) and mostly due to the lack of whole grains and processed meat in the availability-based FBS estimates, both of which resulted in a lower attributable burden.

In an environmental assessment based on life-cycle assessments of environmental footprints, the estimated impacts were similar between the GDD-IA and FBS-based data, but there were large differences between the GDD-IA and survey-based GDD estimates (Fig. 5b and Supplementary Table 14). For example, the survey-based estimates were lower for cropland use (−2.8 million km^2^, −28%) because of an incomplete coverage of food groups, but they were higher for pasture use (+6.2 million km^2^, +27%) because of a greater reported intake of red meat. The GDD-IA and FBS-based estimates are consistent with official statistics on pasture land (~32 million km^2^) (ref. ^17^) when accounting for the additional contributions from food loss and waste (~10 million km^2^), whereas the survey-based ones exceed official statistics (+7.1 million km^2^, +22%). There were additional differences for other environmental indicators and regions (Supplementary Table 13). For example, the GHG emissions associated with food consumption in North America were substantially lower in the survey-based estimates (−290 million tonnes of carbon dioxide equivalent, MtCO_2_eq, −48%) because of a considerably lower intake of red meat reported in the survey data.

In a dietary cost assessment based on market prices of foods, the estimated cost of diets was similar for the GDD-IA and FBS-based data, but they were smaller for the survey-based GDD data (−US$0.6 d^−1^, −12%) because of their incomplete coverage of food groups (Supplementary Fig. 15). There were additional differences across demographic groups (Fig. 5c). The GDD-IA-based estimates indicated a much larger difference in the cost of diets between men and women (+US$0.8 d^−1^, +19%) as the survey-based estimates (+US$0.2 d^−1^, +5%), something that was driven by differences in controlling for energy intake. The GDD-IA normalizes food intake to overall levels of energy intake that correspond to the energy needs of a specific population and that population’s body weight, height and physical activity, whereas the survey-based data of the GDD have been standardized to the same energy intake across women and men (and across all adult ages). In contrast to either, the FBS-based estimates represent national averages and do not allow for differentiating between demographic groups.

## Discussion

Dietary intake has major implications for health, the environment and the economy. However, current global estimates of food intake are fundamentally uncertain and often not regionally comparable. We developed a new dietary database, the GDD-IA, that combines the strengths of existing data sources. For deriving food intake at the national level, we used regionally comparable statistics on food available for human consumption^17^ from which we subtracted waste at retail and household levels^20^ and then normalized to estimates of total energy intake derived from measurements of body height, weight and physical activity^39^. For deriving comparable socio-demographic trends across age groups, sex and urban/rural residence, we used estimates from dietary surveys^32^ and again normalized those to the estimated energy intake of the relevant population group^39^. By construction, our estimates are in line with observed trends in anthropometric measures such as body mass index^42,43^, they include both processed and unprocessed foods^17,32^ and they cover complete diets at global, regional, national and socio-demographic levels.

The GDD-IA addresses the main shortcomings of existing estimates. Compared to waste-adjusted food availability data, the GDD-IA includes estimates for socio-demographic groups within countries, which allows for more detailed analyses and policy planning. The GDD-IA also includes estimates for foods that are particularly relevant for health but not included in food availability data such as whole grains and processed meat. This coverage allows for more comprehensive dietary risk assessments, as illustrated in our example applications. Lastly, normalizing to independent estimates of total energy intake ensures that estimated intake is biologically plausible and implicitly controls for uncertainties in and irregular updates of food waste estimates that affect waste-adjusted food availability data. For example, assuming constant waste fractions as existing impact assessments have done^44^ would overestimate intake if wastage rates have increased (Supplementary Table 6). We therefore think our estimates can inform and be used for more biologically grounded assessments of both food intake and food waste, especially when paired with regular updates of commodity-specific wastage rates^45^.

An alternative to deriving food intake from statistics on food availability could have been to adopt estimated intake from dietary surveys, at least for the foods reported in global databases such as the GDD. However, the variations in survey-based estimates were so wide that they resulted in biophysically implausible intake when combined with estimates of missing foods from our primary proxy, ranging from a population-level energy intake of 1,620 kcal d^−1^ (a level similar to that of children and adolescents) to 3610 kcal d^−1^ (a level similar to that of athletes). An additional inconsistency of the survey-based estimates was that estimates for specific food groups such as red meat exceeded food availability statistics (with waste included) for upper middle-income countries where statistics on food availability are relatively good. The large differences in the reported intake in red meat resulted in estimated environmental impacts that were inconsistent with independent statistics, for example, on pasture use, as illustrated in our example applications. On the basis of these consistency checks, we decided against using dietary surveys for deriving a complete diet proxy and instead used their variation across socio-demographic groups to supplement our main estimates.

A detailed comparison of our primary proxy to one based on dietary surveys indicated large differences. However, most of the differences can be explained by known shortcomings of dietary surveys which have been found to be prone to misreporting due to a variety of factors, including a tendency of respondents to answer according to social norms (social desirability/approval bias)^25,26^, poor understanding and unclear communication of servings sizes^24^ and problems with recalling intake from memory^15^. Specifically, the higher estimated intake from surveys of red meat in middle-income countries could be due to red meat being perceived as high-status foods, whereas the lower estimated intake of roots and legumes in low-income and middle-income countries could be due to these foods being perceived as low-status foods. The lower estimated intake of fruits and vegetables in upper middle-income countries could indicate challenges in understanding and communicating serving sizes, whereas the lower estimated intake of nuts in high-income countries could be due to the use of nuts in processed foods and the consequent challenge in recall.

The comparison also highlighted aspects where our primary proxy could be improved. In particular, the much larger survey-based intake of vegetables, fish and eggs in lower middle-income and low-income countries could be due to a proportion of those foods being produced for own consumption by small-holder farmers or subsistence agriculture, which are not being recorded in food availability statistics (for example, Supplementary Table 6)^39^. One way of incorporating such information is by developing additional proxies based on combining aspects of availability-based and survey-based estimates and by using a larger set of proxies in structured sensitivity analyses. We included an energy-corrected proxy based on dietary surveys in our database to facilitate this (Methods), and we also developed a combined proxy in which we used survey-based estimates for foods that could be from subsistence production in low-income and lower middle-income countries (Supplementary Table 12). In comparison, our main estimates implicitly account for subsistence production by energy correcting the waste-adjusted food availability data, but this implicitly assumes that a proportion of all foods comes from subsistence production.

In addition to the various improvements over existing estimates, our new estimates of food intake are also subject to several caveats. Many of them carry over from those associated with the data sources used in their construction, including estimates of food availability, food waste, estimated energy intake and dietary surveys. Food availability statistics are dependent on the accurate reporting of food balances. Although the FAO has aimed to standardize how food balances are reported and estimated, there is no measure of uncertainty, and the transparency of their methods could be improved, including the exact nature of recent updates that have introduced discontinuities^46^. For our analysis, we harmonized internally consistent estimates of food balances for the years 2010 to 2020 (released up to 2023) with earlier estimates for the years 1990 to 2010 by aligning calorie densities (Methods), and we derived uncertainty ranges by propagating the uncertainty related to the estimates of total energy intake.

Estimates of food waste were last updated by the FAO in 2010/11^20^. Although our method of deriving intake can also be used to derive changes in overall wastage rates over time (by subtracting energy intake from calorie availability), it cannot inform commodity-specific changes apart from those overall shifts. New estimates of food waste have recently been compiled^45^ but not yet standardized in the same way as the FAO’s, which prevented us from using them. For example, they used a different split across the food chain, and their overall estimates resulted in levels of food consumption inconsistent with observed trends in body weight across regions^45^. More frequent and standardized measurements of food waste would improve the estimates of dietary composition in our database.

The estimates of energy intake we used to normalize our estimates were based on the latest equations for estimating energy requirements paired with comprehensive and regionally comparable datasets on body weight, height and physical activity^39^. The uncertainties of these input parameters were propagated through to the final estimates, and the related uncertainty is also the main source of uncertainty of our estimates of food intake. Among the input parameters, body weight and height have been estimated by objective measures, whereas physical activity has been estimated based on surveys which have a greater level of unquantified uncertainty. Efforts are currently underway to use more objective measures for estimating physical activity (for example, accelerometers^47^), which will allow us to more accurately determine overall levels of energy intake.

Ideally, we would have also preferred to use more direct measurements of food intake, but the degree of inconsistencies in dietary-survey estimates made them unsuitable for constructing a consistent and regionally comparable database of food intake that can be used in impact assessments at the population level. Efforts to measure food intake more directly include the use of image-assisted dietary assessments, which can improve the misreporting of serving sizes and incomplete recall^48,49^, and of dietary or recovery biomarkers, which can be used to validate the accuracy and plausibility of reported intake^31,50^. Before more direct and objective estimates of food intake become widely available, critically evaluating and triangulating exiting estimates, as we have done in this study, is one way of addressing existing shortcomings and improving estimates of dietary intake and related assessments.

## Methods

We followed several steps for developing the global dietary database for impact assessments. First, we developed biophysically plausible estimates of primary (that is, unprocessed) food intake at a country level based on normalizing waste-adjusted food availability estimates to estimates of total energy intake. Second, we supplemented the estimates with processing ratios from dietary surveys and supply utilization accounts to derive intake of processed foods. Third, we derived socio-demographic trends in intake from dietary surveys normalized to estimates of total energy intake by population group. We compared our estimates to estimates based on dietary surveys, and we conducted a series of impact assessments to illustrate their uses and advantages over estimates that are based on dietary surveys and food availability data.

### Estimating national-level food intake

We sourced estimates of food available for human consumption from FAO’s Food Balance Sheets (FBS) for the years 1990 to 2020^17^. The estimates are based on country-level data on the production, trade and utilization of food commodities that are obtained annually from official statistics from FAO member countries. They are expressed in primary commodity equivalents (for example, wheat and milk equivalents) and contain estimates for 97 distinct food commodities in 181 countries measured in grams and kilocalories per person per day. Estimates before 2010 are based on a different method for deriving food balances. To avoid discontinuities, we harmonized the calorie densities between the two datasets and re-calculated intake in grams prior to 2010 (Supplementary Section 1), in line recommended practice^46^. For further processing, we aggregated the commodity detail to a list of 36 food groups (Supplementary Table 1).

The FBS data are adjusted for estimated losses during agricultural processing, storage and transportation, but not for food waste occurring at the level of retailers and households. We used the waste-accounting methodology and parameters developed by the FAO to estimate for each commodity and country the amount of food wasted during distribution and consumption^20^ and subtracted those from the estimates of food availability. Supplementary Section 1 and Supplementary Tables 2 and 3 provide a detailed description of the method and parameters used.

This method of deriving a proxy for dietary intake starts at the level of reported values of foods produced, traded and processed and subtracts relevant portions across the food chain on the way to consumption, all of which introduce unquantifiable levels of uncertainties. To ensure estimated intake is biologically plausible, we introduced a further step in our analysis at the level of food consumption. In particular, we normalized food intake to estimates of total energy intake^39^. The latter was based on calculating the energy intake required to sustain measured levels of body weight, height and physical activity. For the normalization, we adjusted estimated food intake by the overall calorie ratio between waste-adjusted availability estimates and the estimates of total energy intake (Supplementary Tables 5 and 6). Supplementary Section 2 provides a detailed description of the estimates of total energy intake.

The normalized estimates of food intake describe intake in terms of primary commodity equivalents (for example, all milk needed to produce the consumed levels of dairy products). Where available, we used processing ratios to supplement the estimates of primary commodities with those of processed foods (adding seven additional food groups). We used FAO’s supply utilization accounts to derive processing ratios that describe the proportion of milk consumed as liquid milk and as processed into dairy products (cheese and yogurt)^17^ and we used GDD estimates from dietary surveys to derive processing ratios for partitioning all grains into refined and whole grains and for total red meat into processed and unprocessed red meat^32^. Because the calorie densities of dairy products differ, we split those according to calories from each products and converted them to the equivalent weights using product-specific calorie densities adapted from the FAO^17^. For grains and red meat, we applied the same processing ratio to weights and calories. Supplementary Table 7 in Supplementary Section 3 provides an overview of the processing ratios used.

### Estimating socio-demographic variation of food intake

We disaggregated the estimates of national food intake derived above by age group, sex and urban/rural residence based on trends in intake we inferred from survey-based estimates and normalized to estimated energy intake per population group. We sourced the survey-based estimates of dietary intake by socio-demographic group from the GDD^32^. The GDD contains estimates of dietary intake of 14 major food groups (along with beverages and nutrients) and stratified by age (in five-year groups), sex (female, male) and residence (urban, rural) for 184 countries and the years 1990–2018. The estimates are based on data from about 1,220 dietary surveys, most of which were nationally representative, covered all age groups and used food-frequency questionnaires or 24-hour recalls^32^. Estimated intake in the GDD has been normalized to a constant level of energy intake per age group (2,000 kcal d^−1^ for ages 11–74, 1,700 kcal d^−1^ for ages 75+ and 6–10 and 700–1,300 kcal d^−1^ for ages 5 and below).

We processed the GDD data in several ways to be able to consistently combine them with our national-level estimates of food intake. To match our time series, we projected the GDD estimates from 2018 to 2020 based on trends in food availability, a method in line with their framework for gap-filling estimates^32^. To align the GDD estimates with biophysically grounded estimates of energy intake, we used the estimates of energy intake that are in line with current biophysical requirements per population group in each country^39^ to re-normalize the GDD estimates to those levels (Supplementary Tables 5 and 6). We then calculated ratios of food intake across the socio-demographic groups for the food groups covered by the GDD (Supplementary Table 8) and mapped those ratios to the food groups covered in our database (Supplementary Table 9). To ensure that the mapping between food groups and source data preserved the overall levels of energy intake, we also normalized the final estimates for each socio-demographic group to their estimated energy intakes. Supplementary Section 4 provides additional descriptions and an overview of the data used in these steps.

The three datasets used to the construct the GDD-IA (waste-adjusted FBS data, GDD data and estimates of energy intake) have global coverage. When a country was not represented in one of the datasets, we dropped that country from our final estimates. This resulted in estimates for 171 countries (Supplementary Table 10).

### Developing additional proxies of food intake

We compared our estimates of food intake to estimates based on dietary surveys. For that, we used the estimates for general food groups contained in the GDD^32^. For comparability, we normalized energy intake to the same estimates of energy intake that we used for our main estimates^39^, and to match the reporting in primary commodity equivalents that is used in our main estimates, we calculated milk equivalents based on the calorie contents of dairy products and the calorie density of milk^17^ and we converted legumes from cooked to dry equivalents based on ratios derived from the US Department of Agriculture nutrition database (FoodData Central). As not all food groups were represented in the GDD, we supplemented them with estimates from our main proxy for missing foods (poultry, sugar, soybeans, vegetable oils, palm and coconut oils, fish oil, butter, cream, poultry, other meat, animal fats, stimulants, spices, alcohol and foods not otherwise classified) and also used our main proxy (in particular, the distribution across specific foods) to disaggregate general food groups into more distinct foods to match the commodity detail of our main estimate (Supplementary Table 11).

For structured sensitivity analysis, we also developed two additional proxies of food intake. In the first, we used dietary-survey estimates from the GDD for informing food composition wherever possible (that is, for the food groups covered by the GDD; Supplementary Table 9), instead of inferring those from waste-adjusted food availability data. The process of construction followed the same steps outlined in the previous paragraph for converting the reporting to primary commodity equivalents, gap-filling missing foods groups and disaggregating general food groups to a more detailed set of commodities. To ensure biological plausibility, we also aligned the uncertainty intervals of food intake to the uncertainty levels of total energy intake that are in line with biophysical requirements at current levels of body weight, height and physical activity levels^39^.

For illustrating how different proxies can be combined, we also developed one proxy in which we supplemented the main FBS-based proxy with GDD-based estimates for foods with a high proportion of subsistence production. On the basis of our comparison between the main proxy and survey estimates, we chose vegetables and fish in low-income and lower middle-income countries as targets for adjustment. We constructed the subsistence-adjusted proxy as follows. First, we adapted the intake values for the targeted foods in the specific regions from the survey-based proxy. Second, we adapted the intake values for the remaining foods and regions from the main proxy. Third, we calculated overall energy intake and normalized it to levels in line with biophysical requirements for consistency. Supplementary Table 12 provides an overview of the additional proxies.

### Illustration in dietary impact assessments

We conducted a series of dietary impact assessment to illustrate the use of the new database of intake estimates and to compare the associated results to those based on other proxies for intake. The impact assessments included a comparative risk assessment of dietary risks, an environmental assessment of the environmental resource use and pollution related to diets and an economic assessment of the cost of diets. We used updated modules of the Dietary Impact Assessment (DIA) model for each of the assessments^51^. Below we provide an overview of the methods used. More detailed descriptions are in the model’s manual^51^ and in dedicated assessments^35,36,52,53^. We chose the year 2015 for this illustration because it was a common year in each of the dietary databases we compared.

The comparative risk assessment included five dietary risk factors and five disease endpoints^35,52^. The risk factors included low intake of fruits, vegetables, legumes, nuts and whole grains and high intake of red meat and processed meat. The disease endpoints included coronary heart disease, stroke, colorectal cancer and type-2 diabetes. The selection of risk-disease associations was supported by available criteria used to judge the certainty of evidence^54–58^. For calculating final health impacts, we used data on cause-specific mortality from the Global Burden of Disease project^59^ and relative risk estimates that relate change in risk factors to changes in disease mortality from meta-analyses of epidemiological cohort studies^54–56,60–62^. We focused on adults aged 20 year or older in our assessment due to low mortality rates of NCDs in younger age groups, and we adjusted the relative risks for attenuation with age based on a pooled analysis of cohort studies focussed on metabolic risk factors, in line with other studies^63^.

For calculating environmental impacts, we paired the estimates of dietary intake with a set of trade-adjusted and regionalized environmental footprints. The environmental footprints were obtained from a comprehensive meta-analysis of life-cycle assessments of 40 foods produced by 38,700 farms in 119 countries, covering GHG emissions, land use, freshwater use, and soil and water pollution as measured by eutrophication potential^64^. The life-cycle assessments have been standardized by harmonizing system boundaries (from inputs to retail) and gap-filling missing steps along the supply chain, which made use of auxiliary estimates (for example, of post-farm processes) and dedicated process-based models (for example, of nitrate leaching)^64^. FAO data on food production and yields have been used to scale to official statistics and regionalize the estimates^17^, and FAO data on trade have been used to derive consumption-based footprints by commodity and region. We multiplied the estimated impacts per person by the population in the specific demographic group^65^ to obtain the overall impacts at the population level.

For calculating the cost of diets, we paired the estimates of dietary intake with estimates of commodity prices^36^. The price estimates were based on a detailed list of commodity prices collected by statistical offices as part of the International Comparison Program led by the World Bank^66^. It included 20,666 estimates of annual average prices in 179 countries, covering 463 food items. The detailed list of food items has been aggregated into the list of general food groups that are represented in the database of dietary intake. For the aggregation, each item has been paired with its caloric content (to control for difference in processing and edible fractions), and averaged prices have been converted from local currency to US$. For the calorie conversion, calorie data from the FoodData Central database maintained by the US Department of Agriculture have been used^67^. The price conversion was based on the application of purchasing-power parity rates to control for differences in prices among countries^66^.

### Reporting summary

Further information on research design is available in the Nature Portfolio Reporting Summary linked to this article.

## Supplementary information

Supplementary Information Supplementary information.

Reporting Summary

Peer Review file

## Data Availability

In line with the FAIR Guiding Principles for scientific data management and stewardship^68^, all results produced in this study are available via Zenodo at 10.5281/zenodo.20818140 (ref. ^40^). They can be downloaded or interactively explored at https://livedataoxford.shinyapps.io/GDD-IA_dashboard/ (ref. ^41^).

## Code availability

The code developed for this study is available via Zenodo at 10.5281/zenodo.20818140 (ref. ^40^).
