# Supplementary material for: Global dietary estimates for conducting health, environmental and economic impact assessments
Source: Nat Food. 2026 Jul 3;7(7):722–32. doi: 10.1038/s43016-026-01388-z (PMC13388106; doi:10.1038/s43016-026-01388-z)
Supplement: Supplementary file 1 — Supplementary information. [file 43016_2026_1388_MOESM1_ESM.pdf]

nature food

Supplementary information https://doi.org/10.1038/s43016-026-01388-z

Global dietary estimates for conducting health, environmental and economic impact assessments

In the format provided by the authors and unedited

Table of Contents

SI1. Adjusting food availability statistics for food waste 2
SI2. Normalising to estimated energy intake 9
SI3. Processed foods 12
SI4. Variation across sociodemographic groups 13
SI.5 Additional proxies 20
SI.6 Supplementary results 22
References 26

SI1. Adjusting food availability statistics for food waste

Food balance sheets (FBS) report on the amount of food that is available for human consumption[1]. They reflect the quantities reaching the market, but do not include waste from both edible and inedible parts of the food commodity occurring at retail and household levels. We first mapped the commodity detail of the food balance sheets to more general food groups (Table S1) and then followed the waste-accounting methodology developed by the FAO[2] to estimate the amount of food wasted at the level of distribution/retail and consumption and subtracted it from the estimates of food availability.

For each commodity and region, we estimated waste-adjusted food availability by multiplying food availability statistics with conversion factors (*cf*) that represent the amount of edible food (e.g. after peeling) (Table S2) and with the percentage of food wasted during distribution (*1-wp(dtr)*) and consumption (*1-wp(cns)*) (Table S3). For roots and tubers, fruits and vegetables, and fish and seafood, we also accounted for the differences in wastage between the proportion that is utilised fresh (pct_{frsh}) and the proportion that is utilised in processed form (pct_{prcd}) (Table S3). The equation used for each food commodity and region was:

$$
\begin{aligned}
&Availability\ adjusted\ for\ waste \\
&\quad = Availability \cdot \frac{pct_{frsh}}{100} \cdot cf_{frsh} \cdot \left(1 - \frac{wp(cns_{frsh})}{100}\right) \cdot \left(1 - \frac{wp(dtr_{frsh})}{100}\right) \\
&\quad + Availability \cdot \frac{pct_{prcd}}{100} \cdot cf_{prcd} \cdot \left(1 - \frac{wp(cns_{prcd})}{100}\right) \cdot \left(1 - \frac{wp(dtr_{prcd})}{100}\right)
\end{aligned}
$$

After adjusting the availability data for waste, we further summarised the estimates. First, we converted sugar cane and sugar beet to refined sugar equivalents by using FAO conversion ratios (0.14 for sugar cane and 0.09 for sugar beet). Second, we allocated offal to the source commodity based on FAO values (10% of offal in beef and lamb each, 6% in pork). Further information about the definition and content of each FBS commodity can be found in the FAOSTAT metadata (https://www.fao.org/faostat/en/#data/FBS).

We expressed intake in grams per person per day (g/d), kilocalories per person per day (kcal/d), and servings per person per day (servings/d). The first two correspond to the units used in the FBS. For the third, we primarily used reference amounts customarily consumed (RACCs) as defined by the US FDA, as well as amounts used in cohort studies (Table S4). Reporting intake in servings is intended for easier of communication. If required, serving sizes can be modified and applied to the estimates in g/d provided in the SI Datafile.

Our analysis combined FBS estimates based on different methodologies[3]. Estimates prior 2010 used a different balancing method which, when compared to estimates post 2010, results in discontinuities in food intake expressed in grams per person per day[4], especially for foods used in composite meals such as milk and sugar. In contrast, the estimates of calorie intake per person and food are more consistent between the two methods. In line with suggested practice[4], we harmonised the estimates prior 2010 by using the estimates of food intake in calories per person to re-calculate food intake in grams per person based on the updated calorie-to-gram ratios. We used the 2010 ratios for this calculation but preserved the annual trends that denote changes in the content of primary commodity equivalents (e.g., the amount of cheese produced from milk).

Table S1. Mapping of food commodities contained in FAO's Food Balance Sheets to food groups used in the analysis.

FAO food group	Aggregated food group
Wheat and products	wheat
Rice and products	rice
Maize and products	maize
Barley and products	othr_grains
Rye and products	othr_grains
Oats	othr_grains
Millet and products	othr_grains
Sorghum and products	othr_grains
Cereals; Other	othr_grains
Potatoes and products	roots
Cassava and products	roots
Sweet potatoes	roots
Roots; Other	roots
Yams	roots
Sugar cane	sugar_cane
Sugar beet	sugar_beet
Sugar non-centrifugal	sugar
Sugar (Raw Equivalent)	sugar
Sweeteners; Other	sugar
Honey	sugar
Beans	legumes
Peas	legumes
Pulses; Other and products	legumes
Soyabeans	soybeans
Nuts and products	nuts
Groundnuts	nuts
Sunflower seed	seeds
Rape and Mustardseed	seeds
Cottonseed	seeds
Sesame seed	seeds

Palm kernels	seeds
Oilcrops; Other	seeds
Soyabean Oil	oil_veg
Groundnut Oil	oil_veg
Sunflowerseed Oil	oil_veg
Rape and Mustard Oil	oil_veg
Cottonseed Oil	oil_veg
Sesameseed Oil	oil_veg
Olive Oil	oil_veg
Ricebran Oil	oil_veg
Maize Germ Oil	oil_veg
Oilcrops Oil; Other	oil_veg
Palmkernel Oil	oil_palm
Palm Oil	oil_palm
Coconut Oil	oil_palm
Tomatoes and products	vegetables
Onions	vegetables
Vegetables; other	vegetables
Oranges; Mandarines	fruits_trop
Lemons; Limes and products	fruits_trop
Grapefruit and products	fruits_trop
Citrus; Other	fruits_trop
Coconuts - Incl Copra	fruits_trop
Pineapples and products	fruits_trop
Dates	fruits_trop
Bananas	fruits_starch
Plantains	fruits_starch
Apples and products	fruits_temp
Grapes and products (excl wine)	fruits_temp
Olives (including preserved)	fruits_temp
Fruits; other	fruits_temp
Coffee and products	stimulants
Cocoa Beans and products	stimulants
Tea (including mate)	stimulants
Pepper	spices
Pimento	spices
Cloves	spices
Spices; Other	spices
Wine	alcohol
Beer	alcohol
Beverages; Fermented	alcohol
Beverages; Alcoholic	alcohol
Bovine Meat	beef
Mutton & Goat Meat	lamb
Pigmeat	pork

Poultry Meat	poultry
Meat; Other	othr_meat
Offals; Edible	offals
Fats; Animals; Raw	fat_ani
Butter; Ghee	butter
cream	cream
Milk - Excluding Butter	milk
eggs	eggs
Freshwater Fish	fish_freshw
Demersal Fish	fish_demrs
Pelagic Fish	fish_pelag
Marine Fish; Other	fish_other
Crustaceans	shellfish
Cephalopods	shellfish
Molluscs; Other	shellfish
Aquatic Animals; Others	fish_other
Aquatic Plants	fish_other
Fish; Body Oil	oil_fish
Fish; Liver Oil	oil_fish
Alcohol; Non-Food	other
Infant food	other
Miscellaneous	other

Notes: Sugar cane and sugar beet were converted (after waste accounting) to sugar equivalents by using FAO conversion ratios (0.14 for sugar cane and 0.09 for sugar beet). Offal was allocated (after waste accounting) to their source commodity based on FAO values (10% of offal in beef and lamb each, 6% in pork). Please see the FAOSTAT metadata for information on the exact content of each FBS commodity (https://www.fao.org/faostat/en/#data/FBS).

Table S2. Conversion factors for determining the edible parts of foods

Food group	Conversion factors	
	by hand	industrial processing
wheat	0.78	N/A
rice	1.00	N/A
maize	0.69	N/A
othr_grains	0.78	N/A
roots	0.74	0.90
vegetables	0.80	0.75
fruits_trop	0.80	0.75
fruits_temp	0.80	0.75
fruits_starch	0.80	0.75
legumes	1.00	N/A
soybeans	1.00	N/A
nuts	0.79	N/A
seeds	0.79	N/A
oil_veg	1.00	N/A
oil_palm	1.00	N/A
sugar_cane	0.78	N/A
sugar_beet	0.78	N/A
sugar	0.78	N/A
stimulants	0.69	N/A
spices	0.69	N/A
alcohol	0.78	N/A
other	0.78	N/A
beef	0.71	N/A
lamb	0.71	N/A
pork	0.68	N/A
poultry	0.73	N/A
othr_meat	0.70	N/A
offals	0.70	N/A
fat_ani	1.00	N/A
butter	1.00	N/A
cream	1.00	N/A
milk	1.00	N/A
eggs	1.00	N/A
fish_freshw	0.50	0.50
fish_demrs	0.50	0.50
fish_pelag	0.50	0.50
fish_other	0.50	0.50
shellfish	0.50	0.50
oil_fish	0.50	0.50

Table S3. Percentage of food wasted during distribution and consumption, and the percentage of processed utilisation.

Food category	Stage in food supply chain	Region						
		Europe	USA, Canda, Oceania	Indus-trialised Asia	Sub-Saharan Africa	North Africa, West and Central Asia	South and South-east Asia	Latin America
cereals	distribution	2	2	2	2	4	2	4
	consumption	25	27	20	1	12	3	10
roots & tubers	percent processed	73	73	15	50	19	10	80
	distribution (fresh)	7	7	9	5	4	11	3
	distribution (prcd)	3	3	3	2	2	8	3
	consumption (fresh)	17	30	10	2	6	3	4
	consumption (prcd)	12	12	12	1	3	5	2
oilseeds & pulses	distribution	1	1	1	2	2	2	2
	consumption	4	4	4	1	2	1	2
fruits & vegetables	percent processed	60	60	4	1	50	5	50
	distribution (fresh)	10	12	8	17	15	10	12
	distribution (prcd)	2	2	2	10	3	10	2
	consumption (fresh)	19	28	15	5	12	7	10
	consumption (prcd)	15	10	8	1	1	1	1
meat	distribution	4	4	6	7	5	7	5
	consumption	11	11	8	2	8	4	6
dairy	distribution	0.5	0.5	0.5	10	8	10	8
	consumption	7	15	5	0.1	2	1	4
eggs	distribution	2	2	4	3	4	3	4
	consumption	8	15	5	1	12	2	4
fish & seafood	percent processed	*40% in low-income countries, 96% for all others*						
	distribution (fresh)	9	9	11	15	10	15	10
	distribution (prcd)	5	5	5	10	5	10	5
	consumption (fresh)	11	33	8	2	4	2	4
	consumption (prcd)	10	10	7	1	2	1	2

Table S4. Serving sizes by food category and food group.

Food category	Food group	Serving size (g)
staples	wheat	45
	rice	45
	maize	45
	other grains	45
	roots	100
fruits&veg	vegetables	100
	tropical fruits	100
	temperate fruits	100
	starchy fruits	100
legumes	legumes	50
	soybeans	50
nuts & seeds	nuts	25
	seeds	25
oils	vegetable oils	15
	palm & coconut oils	15
	fish oil	15
sugar	sugar	8
dairy & fats	milk equivalents	250
	butter	5
	cream	15
	animal fat	15
eggs	eggs	50
red meat	beef	100
	lamb	100
	pork	100
other meat	poultry	100
	other meat	100
fish	freshwater fish	100
	pelagic fish	100
	demersal fish	100
	other fish	100
	shellfish	100
other	stimulants	5
	spices	1.25
	alcohol	140
	other	5
processed foods	whole grains	45
	processed grains	45
	red meat	100
	processed meat	50
	yoghurt	170
	cheese	17.5
	milk	250

SI2. Normalising to estimated energy intake

We normalised the estimates of food intake based on waste-adjusted food availability statistics to estimates of total energy intake based on the energy requirements to sustain measured levels of body weight and heights, and estimated levels of physical activity[5].

The calculations made use of predictive equations for estimating energy requirements (EERs) that have been developed by the Committee on Dietary Reference Intakes for Energy of the US National Academies of Sciences, Engineering, and Medicine (NAS), and released in 2023.[6] The equations are based on estimates of total energy expenditure derived from a database of doubly labelled water (DLW) studies containing 8,600 values including all ages and life stages. They are differentiated by age, sex, and physical activity level, and for each stratum are dependent on age, height, and weight. They were validated against external DLW data that included 5,056 participants.

The data on body weight and height were sourced from the NCD Risk Factor Collaboration (NCD-RisC) which collected and harmonised measurements from population-based studies globally.[7–9] The data are based on 3,663 population-based studies with measurements of weight and height on 22 million participants aged 5 and older. The data are differentiated by age, sex, and urban/rural residence. In the absence of comparable data for years 0 to 5, normative values of weight and height were adopted from WHO's reference growth standards for weight for age and height for age.

The data on physical activity were sourced from a global pooling analysis of surveys on physical inactivity commissioned by the WHO.[10–12] The estimates of physical inactivity were derived from 298 school-based surveys from 146 countries including 1.6 million participants for students, and from 507 surveys across 163 countries including 5.7 million participants for adults. The estimates were made for the prevalence of physical inactivity as defined by the WHO, and in line with the NAS classification. The Prospective Urban and Rural Epidemiology study was used to inform urban-rural splits in physical activity by income region, which was based on data from 698 communities across 22 countries.[13]

The input parameters used for calculating energy requirements, in particular body weight, height and physical inactivity levels, were subject to uncertainty and have been estimated with low and high values of 95% confidence intervals. Standard methods of error propagation (first-order Taylor expansions) were used to incorporate these input-related uncertainties into the final estimates. Table S5 provides an overview of the estimates of total energy intake calculated in that way, and

Table S6 provides an overview of the ratios of energy intake used in the normalisation procedure (i.e., EERs over energy intake in waste-adjusted FBS and the GDD).

Table S5. Estimated energy intake (kcal/d) by demographic groups and regions in 2020. Estimates are reported as mean, low, and high values of 95% confidence intervals.

Grouping	Demographic level	Estimated energy intake (kcal/d)
Global	Average	2158 (2102 - 2213)
Sexes	Females	1925 (1875 - 1976)
	Males	2388 (2327 - 2448)
Age groups	Children	1202 (1153 - 1252)
	Adolescents	2191 (2141 - 2240)
	Adults	2403 (2345 - 2462)
	Young adults	2518 (2460 - 2575)
	Middle-aged adults	2380 (2321 - 2440)
	Senior adults	2100 (2040 - 2161)
Residence	Urban population	2225 (2168 - 2281)
	Rural population	2086 (2021 - 2141)
Income region	High-income countries	2357 (2305 - 2410)
	Upper middle-income	2250 (2200 - 2300)
	Lower middle-income	2027 (1970 - 2083)
	Low-income countries	1976 (1896 - 2057)
Geographic region	North America	2425 (2380 - 2471)
	Latin America & Caribbean	2255 (2195 - 2314)
	Europe & Central Asia	2373 (2311 - 2435)
	Middle East & North Africa	2214 (2145 - 2282)
	East Asia & Pacific	2204 (2157 - 2252)
	South Asia	1995 (1944 - 2045)
	Sub-Saharan Africa	1987 (1917 - 2057)

Table S6. Overview of energy ratios used in the normalisation. They include the ratios between estimated energy requirements to sustain measured levels of body weight, height, and physical activity (EER) and either those from waste-adjusted food availability statistics (FBS) or dietary-survey estimates from the Global Dietary Database (GDD). The ratios differ by country, and the EER/GDD ratio also by sociodemographic group (see Table S5). Estimates are reported as mean, low, and high values of 95% confidence intervals in 2010 and 2020.

Ratio	Region	Value in 2010			Value in 2020		
		mean	low	high	mean	low	high
EER/FBS	WLD	1.00	0.98	1.03	0.97	0.93	1.01
	HIC	1.01	0.99	1.04	0.97	0.94	1.00
	UMC	1.01	0.99	1.03	0.96	0.93	1.00
	LMC	0.98	0.95	1.01	0.95	0.91	0.99
	LIC	1.09	1.04	1.14	1.13	1.08	1.20
EER/GDD	WLD	1.14	1.11	1.17	1.15	1.11	1.20
	HIC	1.24	1.21	1.27	1.25	1.21	1.29
	UMC	1.17	1.14	1.19	1.18	1.15	1.23
	LMC	1.07	1.04	1.10	1.09	1.04	1.13
	LIC	1.10	1.05	1.16	1.12	1.06	1.19

SI3. Processed foods

We calculated the ratio of processed foods from two sources. For processed dairy products, we used the intake values (in kcal/d) reported in FAO's Supply Utilisation Accounts[3] for cheese, yoghurt, and milk to calculate the proportion of calories from each product. We used the calorie densities of each product (also adopted from the Supply Utilisation Accounts) to convert from calories to the equivalent weights in grams. For refined grains and processed meat, we used the intake values (in g/d) reported by the GDD[14], normalised those to the estimated energy intake values reported in SI2[5], and calculated the proportion of refined grains in total grains and of processed meat in total red meat. In absence of regionally comparable calorie densities of refined grains and processed meat (the GDD provides estimates in grams only), we used the same processing ratios for grams and calories, implicitly assuming equal calorie densities between products.

Table S7. Processing ratios of dairy products (% of kcal/d from all dairy), of refined grains (% of g/d from all grains), and of processed meat (% of g/d from all red meat) in 2020.

Food group	Region				
	Global	High-income countries	Upper middle-income	Lower middle-income	Low-income countries
Cheese (% kcal/d)	28%	58%	14%	5%	11%
Yoghurt (% kcal/d)	2%	5%	1%	0%	0%
Refined grains (% g/d)	86%	86%	94%	80%	82%
Processed meat (% g/d)	24%	38%	16%	29%	51%

SI4. Variation across sociodemographic groups

We disaggregated national food intake by age group, sex, and urban/rural residence based on trends in intake inferred from the Global Dietary Database (GDD)[14]. The GDD contains estimates of food intake of 14 food groups derived from individual-level dietary surveys, normalised to a constant level of energy intake per age group (2000 kcal/d for ages 11-74, 1700 kcal/d for ages 75+ and 6-10, and 700-1300 kcal/d for ages 5 and below). We processed the GDD data in several ways to be able to consistently combine them with our national-level estimates of food intake.

To match our time series, we projected the GDD estimates from 2018 to 2020 based on trends in food availability, a method in line with their framework for gap-filling estimates[14]. To align the GDD estimates with biophysically grounded estimates of energy intake, we used the estimates of energy intake that are in line with current biophysical requirements per population group in each country[5] to re-normalise the GDD estimates to those levels (Tables S5-S6). We then calculated ratios of food intake across the socio-demographic groups and for the food groups covered by the GDD (Table S8) and mapped those ratios to the food groups covered in our database (Table S9).

To ensure that the mapping between food groups and source data preserved the overall levels of energy intake, we also normalised the final estimates for each socio-demographic group to their estimated energy intakes[5]. This was necessary because the absolute intake levels for specific food groups in the GDD differ from the absolute levels of intake in the waste-adjusted FBS data, also after each data source is energy-corrected to the same overall level of energy/food intake (see, e.g., Table S11-12). For example, if red-meat intake was greater in the energy-corrected GDD estimates but lower in the energy-corrected FBS-based estimated, then the socio-demographic variation mapped from the former to the latter covered less energy intake, with the remainder of total energy intake covered by other food groups with different coefficients of socio-demographic variation. Thus, the distribution of total energy intake across food groups differs between the GDD-based estimates and the FBS-based ones, which could result in a misalignment of total energy intake when the two sources are mapped between each other. The second normalisation ensures that total food and energy intake in the different socio-demographic groups is consistent in the final estimates and aligns with the external estimates of energy intake (Table S5)[5].

The regional coverage of the source data and of the final estimates is shown in Table S10. The three datasets used to the construct the GDD-IA (FBS data, GDD data, and estimates of energy intake) have global coverage. When a country was not represented in one of the datasets, we dropped that country from our final estimates. This resulted in estimates for 171 countries (Table S10).

Table S8. Overview of variation in intake of selected food groups across sociodemographic groups in 2020. The variation differs by region and also includes the cross sections of age groups, sexes, and residence.

Food group	Age groups					Sexes		Residence	
	0-9	10-19	20-39	40-64	65+	female	male	rural	urban
refined grains	0.52	1.24	1.15	0.99	1.01	0.87	1.13	1.07	0.95
whole grains	0.58	1.11	1.17	1.00	1.02	0.89	1.11	1.06	0.95
potatoes	0.61	1.14	1.19	0.97	0.97	0.87	1.13	0.97	1.02
other roots	0.56	1.45	1.13	0.86	1.02	0.95	1.05	0.90	1.08
fruits	0.51	0.99	1.09	1.10	1.31	0.97	1.03	0.80	1.16
vegetables	0.36	0.92	1.19	1.19	1.14	0.94	1.06	0.96	1.03
legumes	0.42	1.19	1.17	1.01	1.16	0.91	1.09	0.91	1.07
nuts	0.52	0.93	1.17	1.13	1.06	0.89	1.10	0.75	1.20
eggs	0.50	0.96	1.17	1.12	1.07	0.90	1.10	0.78	1.17
milk	0.86	1.23	0.99	0.88	1.24	0.92	1.08	0.75	1.20
yoghurt	0.55	1.06	1.05	1.03	1.44	0.99	1.01	0.60	1.31
cheese	0.41	0.95	1.12	1.12	1.42	0.91	1.08	0.54	1.36
red meat	0.38	1.07	1.23	1.09	1.02	0.88	1.12	0.71	1.23
processed meat	0.60	1.22	1.19	0.94	0.92	0.83	1.17	0.68	1.25
fish	0.36	0.99	1.15	1.16	1.23	0.93	1.07	0.89	1.09

Table S9. Mapping of sociodemographic trends between food groups of the GDD-IA and the GDD.

Food group (GDD-IA)	Food group (GDD)
wheat	grains
rice	grains
maize	grains
othr_grains	grains
roots	roots
vegetables	vegetables
fruits_trop	fruits
fruits_temp	fruits
fruits_starch	fruits
legumes	legumes
soybeans	legumes
nuts	nuts
seeds	nuts
oil_veg	MUFA
oil_palm	SFA
sugar	SSBs
SSBs	SSBs
milk	dairy
eggs	eggs
fish_freshw	fish
fish_pelag	fish
fish_demrs	fish
fish_other	fish
shellfish	fish
poultry	fish
beef	total_red_meat
lamb	total_red_meat
pork	total_red_meat
othr_meat	total_red_meat
stimulants	energy
spices	energy
alcohol	energy
other	energy
fat_ani	SFA
oil_fish	PUFA
butter	dairy
cream	dairy
whole_grains	whole_grains
prc_grains	prc_grains
red_meat	red_meat
prc_meat	prc_meat
milk_actl	milk
yoghurt	yoghurt
cheese	cheese

Table S10. Regional coverage of the global dietary database for impact assessments (GDD-IA) and its data sources, including data from food balance sheets (FBS), the global dietary database (GDD), and the estimates of energy intake in line with requirements (EER). The coverage of source data accounts for the availability of matching population data and is therefore smaller than declared by the respective sources. Countries are denoted by their ISO3 codes.

Country	FBS	GDD	EER	GDD-IA
all	179	171	198	171
AFG			1	
AGO	1	1	1	1
ALB	1	1	1	1
AND			1	
ARE	1	1	1	1
ARG	1	1	1	1
ARM	1	1	1	1
ASM			1	
ATG	1	1	1	1
AUS	1	1	1	1
AUT	1	1	1	1
AZE	1	1	1	1
BDI	1	1	1	1
BEL	1	1	1	1
BEN	1	1	1	1
BFA	1	1	1	1
BGD	1	1	1	1
BGR	1	1	1	1
BHR	1		1	
BHS	1	1	1	1
BIH	1	1	1	1
BLR	1	1	1	1
BLZ	1	1	1	1
BMU			1	
BOL	1	1	1	1
BRA	1	1	1	1
BRB	1	1	1	1
BRN			1	
BTN	1		1	
BWA	1	1	1	1
CAF	1	1	1	1
CAN	1	1	1	1
CHE	1	1	1	1
CHL	1	1	1	1
CHN	1	1	1	1
CIV	1	1	1	1
CMR	1	1	1	1
COD	1	1	1	1
COG	1	1	1	1
COK			1	
COL	1	1	1	1
COM	1	1	1	1
CPV	1	1	1	1

CRI	1	1	1	1
CUB	1	1	1	1
CYP	1	1	1	1
CZE	1	1	1	1
DEU	1	1	1	1
DJI	1	1	1	1
DMA	1	1	1	1
DNK	1	1	1	1
DOM	1	1	1	1
DZA	1	1	1	1
ECU	1	1	1	1
EGY	1	1	1	1
ERI			1	
ESP	1	1	1	1
EST	1	1	1	1
ETH	1	1	1	1
FIN	1	1	1	1
FJI	1	1	1	1
FRA	1	1	1	1
FSM	1		1	
GAB	1	1	1	1
GBR	1	1	1	1
GEO	1	1	1	1
GHA	1	1	1	1
GIN	1	1	1	1
GMB	1	1	1	1
GNB	1	1	1	1
GNQ			1	
GRC	1	1	1	1
GRD	1	1	1	1
GRL			1	
GTM	1	1	1	1
GUY	1	1	1	1
HND	1	1	1	1
HRV	1	1	1	1
HTI	1	1	1	1
HUN	1	1	1	1
IDN	1	1	1	1
IND	1	1	1	1
IRL	1	1	1	1
IRN	1	1	1	1
IRQ	1	1	1	1
ISL	1	1	1	1
ISR	1	1	1	1
ITA	1	1	1	1
JAM	1	1	1	1
JOR	1	1	1	1
JPN	1	1	1	1
KAZ	1	1	1	1
KEN	1	1	1	1
KGZ	1	1	1	1
KHM	1	1	1	1

KIR	1	1	1	1
KNA	1		1	
KOR	1	1	1	1
KWT	1	1	1	1
LAO	1	1	1	1
LBN	1	1	1	1
LBR	1	1	1	1
LBY	1	1	1	1
LCA	1	1	1	1
LKA	1	1	1	1
LSO	1	1	1	1
LTU	1	1	1	1
LUX	1	1	1	1
LVA	1	1	1	1
MAR	1	1	1	1
MDA	1	1	1	1
MDG	1	1	1	1
MDV	1	1	1	1
MEX	1	1	1	1
MHL			1	
MKD	1	1	1	1
MLI	1	1	1	1
MLT	1	1	1	1
MMR	1	1	1	1
MNE	1	1	1	1
MNG	1	1	1	1
MOZ	1	1	1	1
MRT	1	1	1	1
MUS	1	1	1	1
MWI	1	1	1	1
MYS	1	1	1	1
NAM	1	1	1	1
NER	1	1	1	1
NGA	1	1	1	1
NIC	1	1	1	1
NLD	1	1	1	1
NOR	1	1	1	1
NPL	1	1	1	1
NRU	1		1	
NZL	1	1	1	1
OMN	1	1	1	1
PAK	1	1	1	1
PAN	1	1	1	1
PER	1	1	1	1
PHL	1	1	1	1
PLW			1	
PNG	1	1	1	1
POL	1	1	1	1
PRI			1	
PRK	1		1	
PRT	1	1	1	1
PRY	1	1	1	1

PSE			1	
PYF	1		1	
QAT	1		1	
ROU	1	1	1	1
RUS	1	1	1	1
RWA	1	1	1	1
SAU	1	1	1	1
SDN	1	1	1	1
SEN	1	1	1	1
SGP			1	
SLB	1	1	1	1
SLE	1	1	1	1
SLV	1	1	1	1
SOM			1	
SRB	1	1	1	1
STP	1	1	1	1
SUR	1	1	1	1
SVK	1	1	1	1
SVN	1	1	1	1
SWE	1	1	1	1
SWZ	1	1	1	1
SYC	1	1	1	1
SYR	1	1	1	1
TCD	1	1	1	1
TGO	1	1	1	1
THA	1	1	1	1
TJK	1	1	1	1
TKL			1	
TKM	1	1	1	1
TLS	1	1	1	1
TON			1	
TTO	1	1	1	1
TUN	1	1	1	1
TUR	1	1	1	1
TUV			1	
TWN			1	
TZA	1	1	1	1
UGA	1	1	1	1
UKR	1	1	1	1
URY	1	1	1	1
USA	1	1	1	1
UZB	1	1	1	1
VCT	1	1	1	1
VEN	1	1	1	1
VNM	1	1	1	1
VUT	1	1	1	1
WSM	1	1	1	1
YEM	1	1	1	1
ZAF	1	1	1	1
ZMB	1	1	1	1
ZWE	1	1	1	1

SI.5 Additional proxies

Table S11. Overview of estimates of food intake based on survey estimates in 2020. GDD-based estimates were normalised to estimates of energy intake in line with anthropometric measures whilst preserving the GDD-based uncertainty ranges. In addition, food groups were disaggregated based on the compositional detail of waste-adjusted food availability statistics. Gaps in foods not represented in the GDD (poultry, sugar, soybeans, vegetable oils, palm and coconut oils, fish oil, butter, cream, poultry, other meat, animal fats, stimulants, spices, alcohol, and foods not otherwise classified) were filled with values from the main proxy.

Food category	Food group	g/d			kcal/d		
		mean	low	high	mean	low	high
staples	wheat	127	81	244	381	239	734
	rice	183	112	394	450	276	975
	maize	34	24	55	104	75	170
	other grains	22	16	36	66	47	107
	roots	79	46	199	61	35	156
fruits&veg	vegetables	214	190	241	57	51	65
	tropical fruits	28	24	33	13	11	16
	temperate fruits	48	42	56	24	21	28
	starchy fruits	19	16	22	13	11	15
legumes	legumes	10	8	17	34	26	58
	soybeans	4	4	4	13	13	14
nuts & seeds	nuts	8	5	18	31	19	67
	seeds	1	0	2	3	2	10
oils	vegetable oils	20	20	21	196	189	204
	palm & coconut oils	8	8	9	77	74	81
	fish oil	0	0	0	0	0	0
sugar	sugar	43	42	45	139	134	145
dairy & fats	milk equivalents	174	123	312	155	112	270
	butter	3	3	3	25	24	26
	cream	1	1	1	2	2	2
	animal fat	2	2	2	21	20	21
eggs	eggs	24	14	46	35	20	66
red meat	beef	28	18	51	45	29	83
	lamb	7	4	16	14	8	30
	pork	39	30	56	109	84	155
other meat	poultry	25	24	26	36	35	37
	other meat	1	1	1	1	1	2
fish	freshwater fish	13	10	18	9	7	12
	pelagic fish	6	4	9	6	4	9
	demersal fish	4	3	6	3	2	4
	other fish	4	4	6	2	2	3
	shellfish	6	4	7	2	2	3
other	stimulants	2	2	3	3	3	3
	spices	2	2	2	8	7	8
	alcohol	53	51	55	34	33	36
	other	2	1	2	1	1	1
processed foods	whole grains	53	33	102	147	94	281
	processed grains	313	199	628	854	543	1705
	red meat	57	43	77	133	102	177
	processed meat	18	10	46	35	19	90
	yoghurt	21	12	60	16	9	42
	cheese	9	6	22	30	18	73
	milk	102	81	132	99	80	127
total	energy				2173	1618	3613

Table S12. Overview of alternative proxies for food intake (g/d) in 2020. The proxies include the main FBS-based one, one based on GDD estimates where available, and one in which estimates of foods potentially from subsistence production (vegetables and fish in low-income and lower middle-income countries) were used from the GDD-based estimates. For consistency, all proxies were normalised to the same biophysical range of energy intake (Table 1).

Food category	Food group	FBS-based proxy			GDD-based proxy			FBS+GDD(subsistence)		
		mean	low	high	mean	low	high	mean	low	high
staples	wheat	116	112	120	127	123	133	115	111	120
	rice	180	173	186	183	177	190	178	172	185
	maize	33	31	34	34	32	35	32	31	34
	other grains	20	19	21	22	21	23	20	19	20
	roots	136	131	142	79	76	82	134	128	139
fruits&veg	vegetables	236	228	244	214	206	222	255	246	264
	tropical fruits	41	40	43	28	27	29	41	40	43
	temperate fruits	68	66	71	48	47	50	68	65	70
	starchy fruits	30	29	32	19	18	20	30	29	31
legumes	legumes	19	18	20	10	10	10	19	18	20
	soybeans	4	4	4	4	4	4	4	4	4
nuts & seeds	nuts	11	11	12	8	8	9	11	11	11
	seeds	1	1	1	1	1	1	1	1	1
oils	vegetable oils	22	21	23	20	20	21	22	21	23
	palm & coconut oils	7	7	7	8	8	9	7	7	7
	fish oil	0	0	0	0	0	0	0	0	0
sugar	sugar	47	45	49	43	42	45	46	45	48
dairy & fats	milk equivalents	172	165	179	174	167	181	171	165	178
	butter	4	4	4	3	3	3	4	4	4
	cream	1	1	1	1	1	1	1	1	1
	animal fat	2	2	2	2	2	2	2	2	2
eggs	eggs	25	24	26	24	24	25	25	24	26
red meat	beef	17	16	18	28	27	29	17	16	18
	lamb	4	4	4	7	7	7	4	4	4
	pork	24	24	25	39	38	41	24	23	25
other meat	poultry	28	27	29	25	24	26	28	27	29
	other meat	1	1	1	1	1	1	1	1	1
fish	freshwater fish	9	9	10	13	12	13	12	12	13
	pelagic fish	4	4	4	6	6	6	5	5	5
	demersal fish	3	3	3	4	4	4	4	3	4
	other fish	5	5	5	4	4	5	5	5	6
	shellfish	6	6	6	6	5	6	6	6	6
other	stimulants	3	3	3	2	2	3	3	3	3
	spices	3	3	3	2	2	2	3	2	3
	alcohol	53	51	55	53	51	55	52	51	54
	other	2	2	2	2	1	2	2	2	2
processed foods	whole grains	49	47	51	53	51	55	49	47	51
	processed grains	299	288	311	313	302	326	297	287	309
	red meat	34	33	36	57	55	59	34	33	36
	processed meat	11	10	11	18	17	18	11	10	11
	yoghurt	3	3	3	21	20	22	3	3	3
	cheese	8	8	8	9	9	10	8	8	8
	milk	130	125	135	102	98	106	130	125	135

SI.6 Supplementary results

Figure S1. Change in food intake between 1990-2000 (upper panel), 2000-2010 (middle panel), and 2010 and 2020 (lower panel) by food group and income region, age group, sex, and residence. The changes are in each case expressed as ratios to the first year in the interval.

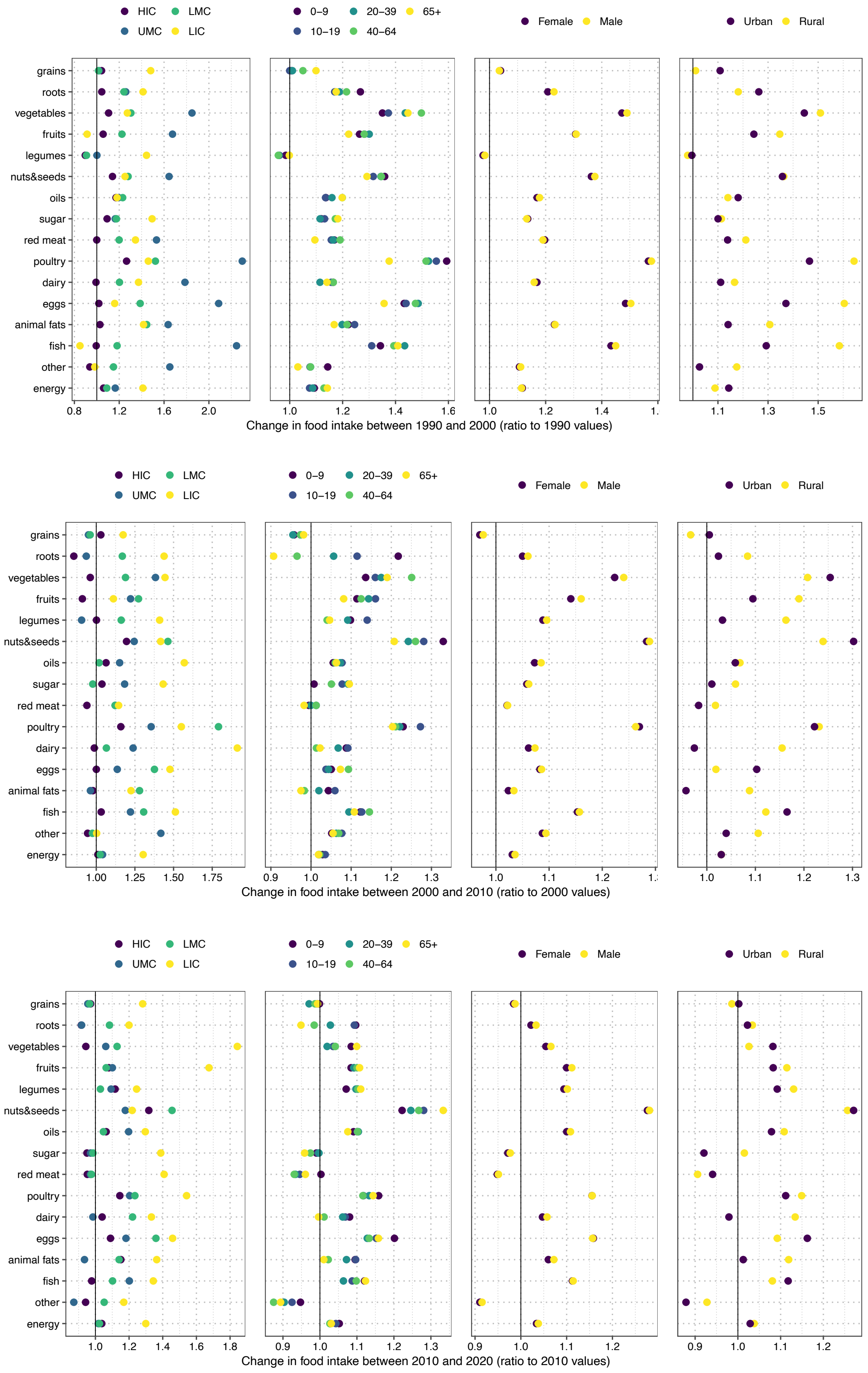

Table S13. Comparison of the estimated number of deaths attributable to dietary risks by dietary data source, and region in 2015. The data sources include the Global Dietary Database for Impact Assessments (GDD-IA), estimates from dietary surveys contained in the Global Dietary Database (GDD), and waste-adjusted food availability data from FAO's food balance sheets (FBS). The regions include the world (WLD), regions grouped by income (high-income, HIC; upper middle-income, UMC; lower middle-income, LMC; low-income, LIC), and regions groups by geography (North America, NAC; Latin America and the Caribbean, LCN; Europe and Central Asia, ECS; the Middle East and North Africa, MEA; South Asia, SAS; East Asia and Pacific, EAS; Sub-Saharan Africa, SSF). The reported values denote the mean, low, and high values of 95% confidence intervals related to the epidemiological uncertainty.

Region	GDD-IA			GDD			FBS		
	mean	low	high	mean	low	high	mean	low	high
WLD	5,803,194	5,428,518	6,178,414	6,773,239	6,311,766	7,235,621	4,841,852	4,470,628	5,213,655
HIC	1,369,235	1,298,520	1,440,045	1,527,753	1,448,159	1,607,478	1,132,621	1,065,367	1,199,943
UMC	2,440,255	2,288,720	2,591,999	2,979,407	2,774,143	3,185,113	1,934,975	1,780,570	2,089,657
LMC	1,777,828	1,642,625	1,913,227	2,022,895	1,866,207	2,179,866	1,562,831	1,433,194	1,692,657
LIC	219,963	202,496	237,450	246,926	226,698	267,186	214,620	194,428	234,842
NAC	380,268	360,593	399,969	449,982	427,626	472,369	289,053	271,471	306,648
LCN	399,634	378,929	420,358	470,544	442,711	498,420	309,277	289,627	328,952
ECS	1,451,254	1,368,719	1,533,937	1,721,452	1,608,304	1,834,997	1,284,798	1,203,748	1,365,983
MEA	239,033	224,443	253,645	305,782	285,567	326,046	188,784	173,690	203,901
SAS	1,093,036	1,004,265	1,181,915	1,198,180	1,099,215	1,297,282	973,234	889,677	1,056,897
EAS	1,910,169	1,787,907	2,032,553	2,293,253	2,143,758	2,442,943	1,509,595	1,381,717	1,637,636
SSF	350,650	323,287	378,045	353,951	323,857	384,083	300,843	273,054	328,675

Table S14. Comparison of the estimated diet-related environmental resource use and pollution by dietary data source, region, and environmental indicator in 2015. The data sources include the Global Dietary Database for Impact Assessments (GDD-IA), estimates from dietary surveys contained in the Global Dietary Database (GDD), and waste-adjusted food availability data from FAO's food balance sheets (FBS). The regions include the world (WLD), regions grouped by income (high-income, HIC; upper middle-income, UMC; lower middle-income, LMC; low-income, LIC), and the example of North America (NAC). The environmental indicators include greenhouse gas emissions (in $GtCO_{2eq}$), cropland use (in million km^2), pasture use (in million km^2), water use (in km^3 divided by 1000), and eutrophication potential (in $MtPO_3^{4-}eq$). The reported values denote the mean, low, and high values of 95% confidence intervals related to the dietary uncertainty (as the environmental footprints did not include uncertainty intervals). The FBS-based estimates have no uncertainty intervals.

Region	Indicator	GDD-IA			GDD			FBS		
		mean	low	high	mean	low	high	mean	low	high
WLD	GHG emissions	10.52	10.21	10.83	9.48	6.45	17.14	10.52		
	cropland use	9.66	9.39	9.95	6.91	4.72	12.51	9.61		
	pasture use	22.94	22.18	23.72	29.11	16.01	57.34	22.12		
	water use	2.18	2.12	2.24	1.99	1.27	4.05	2.21		
	eutrophication	51.22	49.78	52.71	49.13	32.55	92.64	51.56		
HIC	GHG emissions	1.97	1.92	2.03	1.37	1.05	1.96	1.96		
	cropland use	2.17	2.12	2.23	1.25	0.99	1.76	2.16		
	pasture use	4.81	4.70	4.92	6.05	2.81	9.79	4.78		
	water use	0.25	0.25	0.26	0.20	0.15	0.30	0.25		
	eutrophication	8.51	8.29	8.74	6.16	4.76	8.71	8.43		
UMC	GHG emissions	4.91	4.78	5.04	5.31	3.56	9.79	4.97		
	cropland use	3.71	3.62	3.81	3.35	2.19	6.44	3.72		
	pasture use	9.48	9.22	9.74	14.16	9.68	22.74	9.47		
	water use	0.95	0.93	0.97	0.96	0.51	2.47	0.96		
	eutrophication	23.58	22.99	24.18	26.48	16.72	53.69	23.84		
LMC	GHG emissions	3.00	2.90	3.10	2.34	1.57	4.28	3.02		
	cropland use	3.07	2.98	3.17	1.91	1.28	3.50	3.11		
	pasture use	4.96	4.74	5.18	5.12	2.08	12.69	4.65		
	water use	0.87	0.85	0.90	0.75	0.55	1.12	0.90		
	eutrophication	16.69	16.17	17.24	14.35	9.88	24.97	17.10		
LIC	GHG emissions	0.63	0.61	0.66	0.47	0.28	1.11	0.56		
	cropland use	0.71	0.68	0.74	0.40	0.26	0.81	0.63		
	pasture use	3.70	3.52	3.88	3.78	1.44	12.13	3.22		
	water use	0.10	0.10	0.11	0.09	0.06	0.16	0.09		
	eutrophication	2.44	2.34	2.55	2.13	1.19	5.28	2.19		
NAC	GHG emissions	0.61	0.59	0.62	0.32	0.29	0.35	0.64		
	cropland use	0.81	0.79	0.83	0.37	0.33	0.42	0.85		
	pasture use	1.43	1.40	1.46	0.83	0.77	0.89	1.51		
	water use	0.06	0.06	0.06	0.03	0.03	0.03	0.06		
	eutrophication	2.77	2.71	2.84	1.40	1.27	1.56	2.92		

Table S15. Comparison of the estimated cost of diet per person (in US$ per day) by dietary data source, and demographic group in 2015. The data sources include the Global Dietary Database for Impact Assessments (GDD-IA), estimates from dietary surveys contained in the Global Dietary Database (GDD), and waste-adjusted food availability data from FAO's food balance sheets (FBS). The demographic groups include sexes (women, men) and age groups (children aged 0-9, adolescents aged 10-19, young adults aged 20-39, middle-aged adults aged 40-64, and senior adults aged 65 and over). The reported values denote the mean, low, and high values of 95% confidence intervals related to the cost-related uncertainty. The FBS-based estimates are not available for demographic groups.

Demographic group	GDD-IA			GDD			FBS		
	mean	low	high	mean	low	high	mean	low	high
All groups	4.74	3.16	6.39	4.18	2.90	5.48	4.68	3.13	6.29
Women	4.32	2.88	5.82	4.07	2.83	5.33			
Men	5.16	3.44	6.95	4.28	2.97	5.62			
Children	2.44	1.60	3.33	2.48	1.72	3.25			
Adolescents	4.73	3.10	6.44	4.71	3.23	6.22			
Young adults	5.56	3.69	7.51	4.88	3.38	6.41			
Middle-aged adults	5.33	3.60	7.12	4.16	2.91	5.44			
Senior adults	4.80	3.26	6.41	4.05	2.82	5.32			

References

1. Food and Agriculture Organization of the United Nations. *Food Balance Sheets: A Handbook*. (Rome, 2001).
2. Gustavsson, J., Cederberg, C., Sonesson, U., Van Otterdijk, R. & Meybeck, A. *Global Food Losses and Food Waste: Extent, Causes and Prevention*. (2011).
3. Food and Agriculture Organization of the United Nations. *FAOSTAT Statistical Database*. (2022).
4. Vonderschmidt, A., Arendarczyk, B., Jaacks, L. M., Bellows, A. L. & Alexander, P. Analysis combining the multiple FAO food balance sheet datasets needs careful treatment. *The Lancet Planetary Health* **8**, e69–e71 (2024).
5. Springmann, M. Estimates of energy intake, requirements and imbalances based on anthropometric measurements at global, regional and national levels and for sociodemographic groups: a modelling study. *BMJ Public Health* **3**, (2025).
6. *Dietary Reference Intakes for Energy*. (National Academies Press, Washington, D.C., 2023). doi:10.17226/26818.
7. NCD Risk Factor Collaboration (NCD-RisC). A century of trends in adult human height. *eLife* **5**, e13410 (2016).
8. NCD Risk Factor Collaboration (NCD-RisC). Worldwide trends in body-mass index, underweight, overweight, and obesity from 1975 to 2016: a pooled analysis of 2416 population-based measurement studies in 128·9 million children, adolescents, and adults. *Lancet* **390**, 2627–2642 (2017).
9. NCD Risk Factor Collaboration (NCD-RisC). Worldwide trends in underweight and obesity from 1990 to 2022: a pooled analysis of 3663 population-representative studies with 222 million children, adolescents, and adults. *Lancet* **403**, 1027–1050 (2024).
10. Guthold, R., Stevens, G. A., Riley, L. M. & Bull, F. C. Global trends in insufficient physical activity among adolescents: a pooled analysis of 298 population-based surveys with 1·6 million participants. *The Lancet Child & Adolescent Health* **4**, 23–35 (2020).
11. Strain, T. *et al.* National, regional, and global trends in insufficient physical activity among adults from 2000 to 2022: a pooled analysis of 507 population-based surveys with 5·7 million participants. *Lancet Glob Health* **12**, e1232–e1243 (2024).
12. Guthold, R., Stevens, G. A., Riley, L. M. & Bull, F. C. Worldwide trends in insufficient physical activity from 2001 to 2016: a pooled analysis of 358 population-based surveys with 1·9 million participants. *The Lancet Global Health* **6**, e1077–e1086 (2018).
13. Boakye, K. *et al.* Urbanization and physical activity in the global Prospective Urban and Rural Epidemiology study. *Sci Rep* **13**, 290 (2023).

14. Miller, V. *et al.* Global Dietary Database 2017: data availability and gaps on 54 major foods, beverages and nutrients among 5.6 million children and adults from 1220 surveys worldwide. *BMJ Global Health* **6**, e003585 (2021).
