# Supplementary material for: Global dietary estimates for conducting health, environmental and economic impact assessments
Source: Nat Food. 2026 Jul 3;7(7):722–32. doi: 10.1038/s43016-026-01388-z (PMC13388106; doi:10.1038/s43016-026-01388-z)
Supplement: Supplementary file 3 — Peer Review file [file 43016_2026_1388_MOESM3_ESM.pdf]

nature portfolio

Peer Review File

Global dietary estimates for conducting health, environmental, and economic impact assessments

Corresponding Author: Professor Marco Springmann

Version 1:

Reviewer comments:

Reviewer #1

(Remarks to the Author)
Thank you to the authors for the considerable work on this manuscript and underlying data processing and analyses. The paper presents a novel data resource that provides estimates of dietary intake at sub-national levels and across sociodemograhic grouping across the globe, and attempts to adjust for several shortcomings that are inherent in other existing sources of nationally-comparable dietary intake estimates.

The database could be a useful resource for further dietary and nutrition research.

My major concerns are that the GDD-IA data do not seem to be available in the supplementary files for review or exploration, and that many of the methodological descriptions and explanations of how data were processed, and why, are difficult to understand and follow.

Other areas and more specifics regarding where the paper could be strengthened:

General:
- I don't understand the main goal of the paper: is it describing the new database and making it available openly (the authors state the data are in the supplementary datafiles, but these were not made available to this reviewer), or is it simply a quantitative comparison of the (publicly unavailable) database to other existing dietary intake sources?
- Basic descriptive elements of the database and the methods for creating it are missing: for example, how many countries, and which ones, does the GDD-IA have dietary estimates for – and what is completeness of these for all the years between 1990-2020? How did authors deal with missing data sources (i.e., if no dietary intake survey or energy requirements existed for a country to adjust the relevant national FBS data)? Were the energy requirements that are used to adjust the FBS data, country-specific, and where were these sourced from? How did authors align the different food groupings across the FBS, GDD and GDD-IA (92, 14, 37, respectively?)
o A key differentiator of the new GDD-IA is that it adjusts total intake to energy intake requirements differentiated by age, sex, activity level, and country, while the GDD adjusts intake to solely age-specific energy intake. Could the authors compare how their energy intake ranges across these multiple variables differ from the ones used by GDD?

- The authors interchangeably use vague terms to describe the data sources compared in this analysis: for example, "the estimates", "estimated based on dietary surveys", "survey-based estimates", "primary proxy", "one based on dietary surveys", "our estimates", "estimates of food available for human consumption", "main proxy", "different proxies", "one proxy", "survey-based proxy". It is very challenging to follow the logic and what is being referred to across the paper. I would suggest to be direct and simply use GDD-IA, GDD, and FBS, etc., when comparing these data sources.

- The paper needs a re-write for clarity. There are many cases where descriptions of data, data adjustments and data manipulation methods are described in vague terms. Examples:
o L306: "we derived uncertainty ranges by normalising intake to estimates of total energy intake which included uncertainty estimates"
o L319-321: is there a reference for these estimates? Where did all these nationally-specific energy requirements come from?
o L321-323: "the uncertainties of input parameters were propagated through to the final estimates and are also the main parameter that determines the uncertainty in our estimates": it is not clear what is being referred to.
o L398: "we disaggregated national food intake by age group, sex, urban/rural residence inferred from dietary surveys" …. And L400: "we sourced the differences in dietary intake by sociodemographic group from the GDD". Are these two descriptions making a different point, or repeating the same thing?

o L408: what are the authors trying to compare? What is the goal of the several processing iterations of GDD data?
o L409: what is the significant of projecting GDD from 2018 to 2020? What new estimates of energy intake are the authors referring to?
o L420: the goal or rationale for developing this new proxy, or how it is being done, is not clear.
o L432: what do the authors mean by combining proxies? What does this refer to? Why is it being done?
o L472-475: what the authors describe here about adjusting the LCAs is not clear – what specifically is being done to augment/standardize the LCA data, and why? Similarly, L476: "FAO data on food production and yields were used to scale and regionalise estimates" – which estimates are being scaled, and how are FAO data being used to do this?

Results:
- The first indicator described is servings per day of food groups. This is relatively unintuitive (e.g. the authors do not define what a serving of vegetable oil vs. a serving of grains is). I would either remove this and stick to the more intuitive metrics of g/day or kcal/day of food groups, or move the servings/day results to the end of the section.

- What do the ranges mean when referring to an "average of 7.5-8.0 servings per person", or "25-30% less roots and vegetables"?

- Many instances of unclear phrasing:
o Line 119: "changes in food intake were in line with changes in energy intake". Do the authors mean "differences in food intake…"?
o L127: "total energy intake ranged from 7% lower than average in low-income countries…". What is 'average' here? The global average?
o L158: "at the global level, the estimates overlapped with estimates based on dietary surveys for most food groups". Authors need to specify what metric or indicator they are referring to. What does 'overlapped' mean? Are ranges of an indicator overlapping?
o L199: "the survey-based estimates were lower for cropland use". Which two data sources are being compared? What level of diet is being compared here (a global average diet?)
o L202: "the GDD-IA and FBS-based estimates are consistent with official statistics on pasture land" – which country/scale is being compared here in the GDD-IA and FBS? For total global dietary intake?
o L210: "in a dietary cost assessment based on market prices of food, the estimated cost of diet was similar …" – in which country/year, etc?

Discussion:
- A major limitation of this database, because of the standardization to energy intake requirements, is that absolute intake cannot be measured, nor can absolute environmental footprints, dietary costs, etc, be calculated. Over or underconsumption of macronutrients, etc, can also not be assessed. This should be made more clear.

- L240: the GDD-IA addresses the main shortcoming of existing estimates that attempt to be globally comparable (not simply existing dietary estimates … as nationally-representative dietary surveys on their own may be more advantageous than using the GDD-IA as they would allow for estimates of absolute intake). Similarly for L242, national-level policy planning could in many cases be better served by using the national dietary survey, rather than the GDD-IA estimated intake for the same country.

- L257-260: it is unclear what the authors are referring to, or what point is being made. What are "estimates of remaining foods from our primary proxy"?

- L289: what do authors mean by "additional proxies". Are these other iterations of the GDD-IA intake estimates, that use different adjustment methods?

- L291: this description is unclear – what is an energy-corrected proxy based on dietary surveys, and what does it facilitate?

- L304-306: what about data from 2000-2010? Was this harmonized?

- L314: did the authors apply the new estimates of food waste to compare with body weight trends, or is this a citing finds from another paper?

Reviewer #2

(Remarks to the Author)
This manuscript presents a significant and timely advancement in global dietary data. The development of the Global Dietary Database for Impact Assessments (GDD-IA) fills an important methodological gap, and the integration of FAO Food Balance Sheets (FBS) with anthropometric energy-requirement estimates represents a notable innovation. The following comments aim to strengthen clarity, justification, and transparency.

1. Clarify the triangulation strategy
The manuscript notes that dietary surveys such as 24-hour recalls and FFQs "are considered the gold standard in public health research" (lines 47–48), yet immediately highlights substantial misreporting challenges—including serving-size misestimation, recall difficulties, social desirability bias, and systematic under-reporting of energy intake (lines 49–53). This

presents a conceptual inconsistency. If surveys have such well-documented biases, it requires clarification in what sense they serve as a gold standard, and for which aspects of dietary intake this designation applies (absolute levels, food-category composition, or demographic gradients).

This issue carries particular importance because GDD-IA relies on survey-derived relative intake patterns to disaggregate FBS-based national totals across sex, age, and residence. The authors should clarify why these relative differences are assumed to be reliable even when absolute survey intake is not, and whether empirical evidence supports the idea that misreporting biases are proportional—or at least stable—across demographic groups.

More broadly, the manuscript would benefit from discussing existing comparisons between FBS data and high-quality national dietary intake surveys, and explaining why discrepancies are interpreted primarily as survey underestimation rather than potential FBS overestimation. Identifying examples where FBS-normalized intake aligns—or diverges—from rigorous national surveys would help readers understand where GDD-IA is likely to perform well and where uncertainties remain.

2. Provide more detail on how survey-derived demographic scaling works
The demographic scaling procedure is central to GDD-IA, but several steps need additional clarification. The manuscript states that survey-derived relative differences across sex, age, education, and urban/rural residence are applied to disaggregate national totals (lines 100–109). However, given the acknowledged misreporting in dietary surveys (lines 49–53), it is unclear why these demographic ratios should be considered reliable indicators. This concern is particularly relevant for the urban–rural distinction, where survey representativeness and data quality vary widely and misreporting patterns may differ.

Furthermore, many countries have multiple surveys that differ in methodology (24-hour recall, FFQ, HCES) and survey year. The manuscript should clarify how demographic ratios are derived when multiple surveys exist, whether ratios differ across survey types, and how within-country heterogeneity is handled.

It is also unclear how demographic ratios are applied to food groups that are poorly measured or entirely absent in surveys, given that the surveys cover only 14 food groups and 7 beverages while GDD-IA includes 37 food groups. The authors should also explain how demographic scaling is handled in countries without any dietary surveys, as this is essential for understanding global coverage and comparability.

Finally, the manuscript mentions that intake is normalized both before and after applying demographic ratios, but the rationale for this double normalization—and its quantitative effect on the resulting demographic patterns—is not explained. A brief example or schematic would substantially improve transparency.

3. Address subsistence production and informal food sources more directly
Subsistence production, informal food acquisition, and home-produced foods play important roles in many low-income settings, yet they are not fully captured in FBS data. Although the manuscript acknowledges this limitation and proposes an alternative “subsistence-adjusted proxy” for vegetables and fish in low-income and lower-middle-income countries, the adjustment appears narrow and somewhat ad hoc.

It would strengthen the manuscript to more clearly quantify which food groups and regions are most affected by subsistence production, the likely magnitude of underestimation, and whether household consumption and expenditure survey data could be used to calibrate or cross-validate these gaps. Because national totals affect all downstream demographic estimates, more explicit characterization of this uncertainty is important for interpreting GDD-IA, especially in low-income contexts.

4. Reconsider the inclusion of the death-attribution, environmental, and cost demonstrations
The environmental and cost analyses appear intended to illustrate the utility of GDD-IA, but the current demonstrations raise methodological questions and are not as strong as the core contribution. Because GDD-IA and FBS share the same national-level intake totals, their environmental and cost outputs inevitably appear very similar, which limits the interpretive value of the comparisons. In addition, the price data used (retail food prices from the ICP) show considerable within-group variation, making food-group-level cost comparisons difficult to interpret. Similarly, the differences in dietary risk–attributable deaths across GDD-IA, GDD, and FBS appear driven largely by whole-grain intake differences, but this is not fully explained.

For these reasons, the manuscript may be strengthened by removing Figure 5 or simplifying it substantially, accompanied by a concise statement noting that GDD-IA can support downstream applications without presenting detailed examples that are difficult to interpret and not central to the methodological advance.

Reviewer #3

(Remarks to the Author)
INTRODUCTION
The authors have done a great job in summarizing the problem statement, the positioning of this study and its contribution to the literature. I do not have specific remarks on the introduction.

RESULTS
The results section is well structured and provides an overview of the constructed database. To enrich its message, I would

advise the authors to integrate an additional short paragraph where country- or region-specific estimates of total food intakes are reported and described, illustrating the variation in total calorie intakes. Something you have now in Figure 1b but that is missing in the main text.
I believe more explanation is needed for Figure 2. The results by gender and residence appear to be specular across the two categories (male-females, rural-urban). Is this by construction of the used variable? Please add a couple of sentences to explain why is that so and comment on the findings.
Lines 163-165: I believe it is necessary to further explain gap-filling procedure in detail. This comment also applies to the methodology or SI section where I think you should expand on the description of such procedure.

DISCUSSION
I completely miss a comparison with currently available databases and an explanation of why the provided estimates in this manuscript align or differ with the current literature. This also applies for the amount of country-level waste calculations, which seems to be key in obtaining the final estimates of net food intakes.

On top of this, I would like to see a reflection on the methodology you used to assess the amount of nutrients embedded in processed foods, especially providing the conversion factors used.

Line 232-235: As the survey has a more limited coverage of food groups may you further explain how this procedure was performed? Additionally, how does this relate or cope with the differences/inconsistencies in the surveys estimates remarked in the introduction and results section?

Line 261-264: An additional inconsistency of the survey-based estimates was that estimates for specific food groups such as red meat exceeded food-availability statistics (with waste included) for upper middle-income countries where statistics on food availability are relatively good.
How do the authors deal with this potential inconsistency?

Lines 309-312: Although our method of deriving intake can also be used to derive changes in overall wastage rates over time (by subtracting energy intake from calorie availability), it cannot inform commodity-specific changes.
I do not comprehend why you state that you cannot address commodity-specific changes of waste rates over time. In principle you have gross intakes by commodity (FBS) and commodity-specific waste rates (Gustavsson et al) so why couldn't you calculate this?

Lines 312-216: New estimates of food waste have recently been compiled, but not yet standardized in the same way as the FAO's, which prevented us from using them. For example, they used a different split across the food chain, and their overall estimates resulted in levels of food consumption inconsistent with observed trends in body weight across regions.
The used food waste fractions from FAO (Gustavsson et al., 2011) have been widely criticized and addressed as inconsistent by FAO itself (FAO, 2019). As stated by the authors, more recent waste fractions are available from the literature (e.g. Gatto and Chepeliev, 2024). Such estimates provide the same framework of Gustavsson (share of lost or wasted food by region, food type, and supply chain stage), overcoming however the much discussed limitations of Gustavsson et al, (i.e. non-food use of food product considered as waste and FLW shares computed only on edible part of food commodities). The split across the supply chain adopted by Gatto and Chepeliev is the same of Gustavsson et al. Net intake levels derived by applying Gatto and Chepeliev's food waste shares remain within the plausible range of daily calorie intakes (as illustrated in the SI of their publication). For this, as this estimates are a collection of waste fractions derived from more recent studies, I encourage the authors to test the application of this more up-to-date waste fractions, with the aim of improving the accounting of food waste across countries.

METHODOLOGY
A general remark regards the application of waste fractions. It is unclear if the authors apply a total waste fraction to overall intakes by country or if this procedure is done at the commodity level. In principle, given the amount of information from the FBS, Gustavsson et al., you should be able to apply commodity-specific waste fractions to commodity-specific intakes. If you don't do so, please explain why. Generally, please add a more detailed description of this procedure. If a total waste fraction is adopted, please describe the method used to calculate the average waste fraction across commodities.
Lines 347-349: How does this step deal/account for the inconsistency of dietary surveys across specific commodities outlined across the paper?
Please illustrate (in the SI) how you calculate how much is consumed directly and how much through all processed foods that are not reported in the SUA? Like Infant food or miscellaneous. In this, please list all processed foods accounted in this calculations.
Lines 391-392: How about other processed foods? How are they treated?
Line 432: Please use either “To illustrate” or “For illustrating” .

SUPPLEMENTARY INFORMATION
Please add an overview of country-specific food waste levels calculated in this process.
Please list the waste fractions used by food product and the food intake estimates before and after the food waste fractions are applied.

Table S3. Where do this percentages of processed foods in overall food consumption come from?

Version 2:

Reviewer comments:

Reviewer #1

(Remarks to the Author)
Thanks to the authors for addressing the feedback, making revisions, adding details to the manuscript, and/or clarifying our questions. Some additional comments for consideration:

- "The most commonly used data in impact assessments are estimates of how much food is available in a country": this may well be true, but just wanted to check - are these quantifiably the most commonly used, or just commonly used?

- I still think the terminology referring to the various datasets and underlying resources is unclear. There are close to 150 instances of 'estimates' or 'the estimates' in the paper. The authors replied that they "provide a description linking these terms to their abbreviations in the first results section", though that section does not provide that - there is no standard set of definitions and abbreviations. The paper still uses a variety of terms to refer to a single dataset.

For example, references to the GDD include: survey estimates, estimates based on dietary surveys, survey-based data, global estimates of food intake, estimated intake from dietary surveys, intake from surveys

References to FAO FBS include: Food availability statistics, availability-based, food availability data, estimates of food availability, national estimates, availability estimates, food balances, estimates of food balances, FBS-based estimates.

Please consider using simplified and consistent terms like GDD, GDD-IA and FBS. This will reduce word count, clarify which dataset is being referred to (e.g. instances of 'national estimates' that I think refer to FBS data, but this could refer to any of the various data that are at national level). It is also helpful as it differentiates when authors refer to survey/data types generally (when they discuss biases or strengths of a certain data type or survey format) versus when they are referring to the specific GDD/FAO databases. Again, all of this would provide a smoother, more coherent reading experience.

- Please consider differentiating and making more clear, what is an existing data resource versus a data manipulation/adjustment process that authors conducted. Examples:

“Estimated intake in the GDD was normalised to a constant level of energy intake” [I assume this already the characteristic of that dataset, rather than authors conducting this as a step. Simply changing to "is normalised", or similar, would help clarify]

"The LCAs were standardised by harmonising system boundaries (from inputs to retail) and gap-filling missing steps along the supply chain, which made use of auxiliary estimates (e.g., of post farm processes) and dedicated process-based models (e.g., of nitrate leaching). FAO data on food production and yields were used to scale to official statistics and regionalise the estimates, and FAO data on trade were used to derive consumption-based footprints by 16 538 commodity and region” [In the reviewer replies, the authors explained that they are just describing the Poore/Nemececk data ... but the way it is worded is indistinguishable from the many other data adjustment steps the authors describe they conducted]

- This specific example here may be a combination of the considerations I have above:

“We disaggregated national food intake by age group, sex, and urban/rural residence based on trends in intake inferred from dietary surveys and normalised to estimated energy intake per population group. We sourced the differences in dietary intake by sociodemographic group from the GDD.”

Reading this, I'm confused on what step is being described in the data adjustment (apart from the energy intake normalisation). The GDD already has intake disagregated by age group, sex, urban/rural residence. Does "national food intake" refer to the starting database (if so, it would be simpler to say GDD or FBS), or does it just refer generally to data at a national level. The phrase "intake inferred from dietary surveys" is already shorthand that the authors used the GDD (so would be simpler to say this), and would not need to repeat the same point again in the next sentence "We sourced the differences in dietary intake by sociodemographic group from the GDD." This is a small example, but many of these around the paper.

I hope these are constructive, and would help with reproducibility of the impressive analysis by others.

Reviewer #2

(Remarks to the Author)
Thank you for the thorough and well-considered responses to my comments. The revisions have addressed all of my questions satisfactorily. Congratulations on a strong academic contribution and an excellent dataset.

Reviewer #3

(Remarks to the Author)
Based on the authors' comprehensive point-by-point responses and the subsequent revisions to the manuscript, I am satisfied with the technical clarifications provided. The authors have effectively addressed my concerns regarding the transparency of the gap-filling procedures and the biophysical grounding of their energy intake estimates. I particularly appreciate the nuanced explanation regarding the symmetry of socio-demographic trends—linking them to physiological requirements and dietary transitions—which clarifies and enriches the overall methodology of the manuscript. Furthermore, the decision to prioritize consistency with FAO food balances over more recent, yet potentially inconsistent, waste datasets (e.g., Gatto and Chepeliev) is well-justified and maintains the internal logic of the study. The addition of absolute energy intake values and the provision of country-specific data via an external repository significantly enhance the paper's utility and reproducibility.

Overall, the authors have successfully refined the manuscript to meet high standards, and I fully recommend this paper for publication.

Version 3:

Reviewer comments:

Reviewer #1

(Remarks to the Author)
I have no further comments. Thank you to the authors for incorporating all feedback.

Open Access This Peer Review File is licensed under a Creative Commons Attribution 4.0 International License, which permits use, sharing, adaptation, distribution and reproduction in any medium or format, as long as you give appropriate credit to the original author(s) and the source, provide a link to the Creative Commons license, and indicate if changes were made.
In cases where reviewers are anonymous, credit should be given to 'Anonymous Referee' and the source.
The images or other third party material in this Peer Review File are included in the article's Creative Commons license, unless indicated otherwise in a credit line to the material. If material is not included in the article's Creative Commons license and your intended use is not permitted by statutory regulation or exceeds the permitted use, you will need to obtain permission directly from the copyright holder.
To view a copy of this license, visit https://creativecommons.org/licenses/by/4.0/

INTRODUCTION

The authors have done a great job in summarizing the problem statement, the positioning of this study and its contribution to the literature. I do not have specific remarks on the introduction.

RESULTS

The results section is well structured and provides an overview of the constructed database. To enrich its message, I would advise the authors to integrate an additional short paragraph where country- or region-specific estimates of total food intakes are reported and described, illustrating the variation in total calorie intakes. Something you have now in Figure 1b but that is missing in the main text.

I believe more explanation is needed for Figure 2. The results by gender and residence appear to be specular across the two categories (male-females, rural-urban). Is this by construction of the used variable? Please add a couple of sentences to explain why is that so and comment on the findings.

Lines 163-165: I believe it is necessary to further explain gap-filling procedure in detail. This comment also applies to the methodology or SI section where I think you should expand on the description of such procedure.

DISCUSSION

I completely miss a comparison with currently available databases and an explanation of why the provided estimates in this manuscript align or differ with the current literature. This also applies for the amount of country-level waste calculations, which seems to be key in obtaining the final estimates of net food intakes.

On top of this, I would like to see a reflection on the methodology you used to assess the amount of nutrients embedded in processed foods, especially providing the conversion factors used.

Line 232-235: As the survey has a more limited coverage of food groups may you further explain how this procedure was performed? Additionally, how does this relate or cope with the differences/inconsistencies in the surveys estimates remarked in the introduction and results section?

Line 261-264: *An additional inconsistency of the survey-based estimates was that estimates for specific food groups such as red meat exceeded food-availability*

statistics (with waste included) for upper middle-income countries where statistics on food availability are relatively good.

How do the authors deal with this potential inconsistency?

Lines 309-312: *Although our method of deriving intake can also be used to derive changes in overall wastage rates over time (by subtracting energy intake from calorie availability), it cannot inform commodity-specific changes.*

I do not comprehend why you state that you cannot address commodity-specific changes of waste rates over time. In principle you have gross intakes by commodity (FBS) and commodity-specific waste rates (Gustavsson et al) so why couldn't you calculate this?

Lines 312-216: *New estimates of food waste have recently been compiled, but not yet standardized in the same way as the FAO's, which prevented us from using them. For example, they used a different split across the food chain, and their overall estimates resulted in levels of food consumption inconsistent with observed trends in body weight across regions.*

The used food waste fractions from FAO (Gustavsson et al., 2011) have been widely criticized and addressed as inconsistent by FAO itself (FAO, 2019). As stated by the authors, more recent waste fractions are available from the literature (e.g. Gatto and Chepeliev, 2024). Such estimates provide the same framework of Gustavsson (share of lost or wasted food by region, food type, and supply chain stage), overcoming however the much discussed limitations of Gustavsson et al, (i.e. non-food use of food product considered as waste and FLW shares computed only on edible part of food commodities). The split across the supply chain adopted by Gatto and Chepeliev is the same of Gustavsson et al. Net intake levels derived by applying Gatto and Chepeliev's food waste shares remain within the plausible range of daily calorie intakes (as illustrated in the SI of their publication). For this, as this estimates are a collection of waste fractions derived from more recent studies, I encourage the authors to test the application of this more up-to-date waste fractions, with the aim of improving the accounting of food waste across countries.

METHODOLOGY

A general remark regards the application of waste fractions. It is unclear if the authors apply a total waste fraction to overall intakes by country or if this procedure is done at the commodity level. In principle, given the amount of information from the FBS, Gustavsson et al., you should be able to apply commodity-specific waste fractions to commodity-specific intakes. If you don't do so, please explain why. Generally, please add a more detailed description of this procedure. If a total waste fraction is adopted, please describe the method used to calculate the average waste fraction across commodities.

Lines 347-349: How does this step deal/account for the inconsistency of dietary surveys across specific commodities outlined across the paper?

Please illustrate (in the SI) how you calculate how much is consumed directly and how much through all processed foods that are not reported in the SUA? Like Infant food or miscellaneous. In this, please list all processed foods accounted in this calculations.

Lines 391-392: How about other processed foods? How are they treated?

Line 432: Please use either "To illustrate" or "For illustrating" .

SUPPLEMENTARY INFORMATION

Please add an overview of country-specific food waste levels calculated in this process.

Please list the waste fractions used by food product and the food intake estimates before and after the food waste fractions are applied.

Table S3. Where do this percentages of processed foods in overall food consumption come from?

Dear editor and reviewers,

Thank you for your comments and suggestions on our manuscript. We have thoroughly revised the manuscript in response and, among other things, substantially clarified and extended our description of methods. The following point-by-point response details the changes made.

<u>Response to Reviewer #1</u>

Comment 1-0:
Thank you to the authors for the considerable work on this manuscript and underlying data processing and analyses. The paper presents a novel data resource that provides estimates of dietary intake at sub-national levels and across sociodemograhic grouping across the globe, and attempts to adjust for several shortcomings that are inherent in other existing sources of nationally-comparable dietary intake estimates.

The database could be a useful resource for further dietary and nutrition research.

Reply to Comment 1-0:
Thanks a lot for your helpful comments and suggestions. Please find our point-by-point response below.

Comment 1-1:
My major concerns are that the GDD-IA data do not seem to be available in the supplementary files for review or exploration, and that many of the methodological descriptions and explanations of how data were processed, and why, are difficult to understand and follow.

Reply to Comment 1-1:
Thank you for the comment. We will make the full database available upon publication. Following your comment, we uploaded the raw data to an online folder: https://drive.google.com/drive/folders/1XF2btDT3sPyuz9Iv3pXinvuGtIkCDWcP?usp=sharing . We are currently working on integrating the data into an interactive analysis tool. Following your comments, we also expanded the description of our methods. We detail them in response to your specific comments below.

Comment 1-2:
Other areas and more specifics regarding where the paper could be strengthened:
General:
- I don't understand the main goal of the paper: is it describing the new database and making it available openly (the authors state the data are in the supplementary datafiles, but these were not made available to this reviewer), or is it simply a quantitative comparison of the (publicly unavailable) database to other existing dietary intake sources?

Reply to Comment 1-2:
The goal of the paper is to present the new database. It's classified as resource paper (a new category) by the journal. We describe the topic of the paper as follows:

In the abstract:

> "Here we develop a new Global Dietary Database for Impact Assessments"

In the introduction:

> "Here we develop a new global dietary database that is particularly designed for use in impact assessments [..].

Below, we provide an overview of the intake estimates, compare them to estimates from dietary surveys, and illustrate how they improve health, environmental, and economic impact assessments related to diets."

And in the discussion section:

> "We developed a new Global Dietary Database for Impact Assessments (GDD-IA) that combines the strengths of existing data sources."

Comment 1-3:
- Basic descriptive elements of the database and the methods for creating it are missing: for example, how many countries, and which ones, does the GDD-IA have dietary estimates for – and what is completeness of these for all the years between 1990-2020? How did authors deal with missing data sources (i.e., if no dietary intake survey or energy requirements existed for a country to adjust the relevant national FBS data)? Were the energy requirements that are used to adjust the FBS data, country-specific, and where were these sourced from? How did authors align the different food groupings across the FBS, GDD and GDD-IA (92, 14, 37, respectively?)

Reply to Comment 1-3:
Thanks for these questions. The number of countries and the mapping between food groups are detailed in the methods section as follows:

> "[The Food Balance Sheets] contain estimates for 97 distinct food commodities in 181 countries [..]. For further processing, we aggregated the commodity detail to a list of 37 food groups (Table S1).
>
> [The GDD] contains estimates of dietary intake of 14 major food groups [..] for 184 countries [..]. [W]e mapped those [..] to the food groups covered in our database (Table S9)"

The estimates of energy intake are described in and were taken from a dedicated paper (reference 39 in the initial manuscript):

> Springmann, M. Estimates of energy intake, requirements and imbalances based on anthropometric measurements at global, regional and national levels and for sociodemographic groups: a modelling study. *BMJ Public Health* 3, (2025).

We added a description of the methods followed in this paper in section SI2 of the supplementary information which we refer to from the main methods section as follows:

> "To ensure estimated intake is biologically plausible, we introduced a further step in our analysis at the level of food consumption. In particular, we normalised food intake to estimates of total energy intake[39]. The later was based on calculating the energy intake required to sustain measured levels of body weight, height, and physical activity. For the normalisation, we adjusted estimated food intake by the overall calorie ratio between waste-adjusted availability estimates and the estimates of total

> energy intake (Tables S5-S6). Section SI2 provides a detailed description of the estimates of total energy intake."

Our approach to missing data was as follows: when there were countries for which we did not have estimates from any of the three sources used to construct the GDD-IA, we dropped that country. This resulted in a coverage of 171 countries. Following your comment, we now inserted a list of countries and their data coverage in the supplementary information (new Table S10), and we added a description and reference to this in the main methods section which reads as follows:

> "The three datasets used to the construct the GDD-IA (FBS data, GDD data, and estimates of energy intake) have global coverage. When a country was not represented in one of the datasets, we dropped that country from our final estimates. This resulted in estimates for 171 countries (Table S10)."

Comment 1-4:
o A key differentiator of the new GDD-IA is that it adjusts total intake to energy intake requirements differentiated by age, sex, activity level, and country, while the GDD adjusts intake to solely age-specific energy intake. Could the authors compare how their energy intake ranges across these multiple variables differ from the ones used by GDD?

Reply to Comment 1-4:
Sure. An overview of our estimates of energy intake is provided in Table S5 in the SI. The estimates differ for each population group in each country based on the population's weight, height, and physical activity levels. They are estimates of energy intake in the sense that these levels of energy intake are required to support the levels of body weight, height, and physical activity of each population group. In contrast, the GDD normalised energy intake to the same energy intake in each age group in all countries. The concrete levels are described in the main methods section as follows:

> "Estimated intake in the GDD was normalised to a constant level of energy intake per age group (2000 kcal/d for ages 11-74, 1700 kcal/d for ages 75+ and 6-10, and 700-1300 kcal/d for ages 5 and below)."

Comment 1-5:
- The authors interchangeably use vague terms to describe the data sources compared in this analysis: for example, "the estimates", "estimated based on dietary surveys", "survey-based estimates", "primary proxy", "one based on dietary surveys", "our estimates", "estimates of food available for human consumption", "main proxy", "different proxies", "one proxy", "survey-based proxy". It is very challenging to follow the logic and what is being referred to across the paper. I would suggest to be direct and simply use GDD-IA, GDD, and FBS, etc., when comparing these data sources.

Reply to Comment 1-5:
Thank you for that comment. Generally, we try to avoid the use of abbreviations to increase readability. For that reason, we prefer descriptive terms such as survey-based estimates for the GDD data, estimates of food available for human consumption for the FBS data, and

main or primary proxy for the main estimates of the GDD-IA. We provide a description linking these terms to their abbreviations in the first results section which reads as follows:

> "The data sources used to construct the GDD-IA differed in their coverage of food groups and sociodemographic details (Figure 1). The data on food available for human consumption from FAO's Food Balance Sheets (FBS) covered 92 food commodities and five beverages by country for the years 1990 to 2020 (with estimates reaching back to 1961). The data on reported intake from dietary surveys included in the GDD covered 14 food groups and 7 beverages by country, age, sex, education, and urban/rural residence for the years 1990 to 2018. The data on estimated energy requirements (EER) covered total energy intake by country, age, sex, and urban/rural residence for the years 1990 to 2020. We combined and harmonised the data (Methods) to cover food intake (in grams, kilocalories, and servings per person and day) for 37 food groups by country, age, sex, and urban/rural residence for every five years between 1990 and 2020 (Table 1)."

Based on this exposition, we think it is clear in each case which data is meant, and that using descriptive terms is easier to understand for most readers than using abbreviations. Please do let us know though if there are specific cases where you felt this was not the case.

Comment 1-6:
- The paper needs a re-write for clarity. There are many cases where descriptions of data, data adjustments and data manipulation methods are described in vague terms. Examples:
o L306: "we derived uncertainty ranges by normalising intake to estimates of total energy intake which included uncertainty estimates"
o L319-321: is there a reference for these estimates? Where did all these nationally-specific energy requirements come from?
o L321-323: "the uncertainties of input parameters were propagated through to the final estimates and are also the main parameter that determines the uncertainty in our estimates": it is not clear what is being referred to.
o L398: "we disaggregated national food intake by age group, sex, urban/rural residence inferred from dietary surveys" …. And L400: "we sourced the differences in dietary intake by sociodemographic group from the GDD". Are these two descriptions making a different point, or repeating the same thing?
o L408: what are the authors trying to compare? What is the goal of the several processing iterations of GDD data?
o L409: what is the significant of projecting GDD from 2018 to 2020? What new estimates of energy intake are the authors referring to?
o L420: the goal or rationale for developing this new proxy, or how it is being done, is not clear.
o L432: what do the authors mean by combining proxies? What does this refer to? Why is it being done?
o L472-475: what the authors describe here about adjusting the LCAs is not clear – what specifically is being done to augment/standardize the LCA data, and why? Similarly, L476: "FAO data on food production and yields were used to scale and regionalise estimates" – which estimates are being scaled, and how are FAO data being used to do this?

Reply to Comment 1-6:
Thank you for your questions and suggestions. We addressed each one in turn.

o L306: "we derived uncertainty ranges by normalising intake to estimates of total energy intake which included uncertainty estimates"

Here we meant that we error-propagated the uncertainty estimates from those related to energy intake. We rewrote this sentence accordingly:

> "we derived uncertainty ranges by propagating the uncertainty related to the estimates of total energy intake"

o L319-321: is there a reference for these estimates? Where did all these nationally-specific energy requirements come from?

Yes, we referenced the source in the introduction and methods (ref 39 in the original manuscript), and inserted it again now following your comment:

> "The estimates of energy intake we used to normalise our estimates were based on the latest equations for estimating energy requirements paired with comprehensive and regionally comparable datasets on body weight, height, and physical activity[39]."

o L321-323: "the uncertainties of input parameters were propagated through to the final estimates and are also the main parameter that determines the uncertainty in our estimates": it is not clear what is being referred to.

The previous sentence mentions the input parameters for deriving energy intake (body weight, height, physical activity). The uncertainties of these data were propagated through to the final estimates of energy intake (error propagation is a standard method of uncertainty analysis that we don't think requires additional explanation). As noted earlier, the uncertainty related to energy intake is the main source of uncertainty for the estimates of food intake, but we could have formulated this more clearly. Following your comment, we clarified in the sentence which input parameters we meant, and that the uncertainty related to energy intake is the main source of uncertainty of food intake:

> "The uncertainties of these input parameters [referring to the "regionally comparable datasets on body weight, height, and physical activity" mentioned in the previous sentence] were propagated through to the final estimates and the related uncertainty [the uncertainty related to energy intake] is also the main source of uncertainty of our estimates of food intake."

o L398: "we disaggregated national food intake by age group, sex, urban/rural residence inferred from dietary surveys" …. And L400: "we sourced the differences in dietary intake by sociodemographic group from the GDD". Are these two descriptions making a different point, or repeating the same thing?

The first sentence is the introductory sentence of the paragraph and therefore explains the overall procedure. The second sentence described where the specific data for inferring the socio-demographic trends came from.

o L408: what are the authors trying to compare? What is the goal of the several processing iterations of GDD data?

As the introductory sentence of this paragraph explains, the GDD data had to be processed in several ways to make them comparable (and combinable) with the other data. Specifically, we wanted to produce a time series up to 2020 to match the other data sources, for which we had to project forward the GDD data from 2018 to 2020. We also wanted to be able to inform trends across socio-demographic groups based on their actual intake instead of the uniform nomalisation used by the GDD, for which we had to adjust the GDD's normalisation procedure. Lastly, we had to map the food groups of the GDD to those of the FBS data as the former contained more general food groups compared to the more detailed ones of the FBS. Following your comment, we edited the paragraph to be clearer on these points. It now states:

> "We processed the GDD data in several ways to be able to consistently combine them with our national-level estimates of food intake. To match our time series, we projected the GDD estimates from 2018 to 2020 based on trends in food availability, a method in line with their framework for gap-filling estimates[32]. To align the GDD estimates with biophysically grounded estimates of energy intake, we used the estimates of energy intake that are in line with current biophysical requirements per population group in each country[39] to re-normalise the GDD estimates to those levels (Tables S5-S6). We then calculated ratios of food intake across the socio-demographic groups and for the food groups covered by the GDD (Table S8) and mapped those ratios to the food groups covered in our database (Table S9). To ensure that the mapping between food groups and source data preserved the overall levels of energy intake, we also normalised the final estimates for each socio-demographic group to their estimated energy intakes. Section SI4 provides additional descriptions and an overview of the data used in these steps."

o L409: what is the significant of projecting GDD from 2018 to 2020? What new estimates of energy intake are the authors referring to?

We needed to project forward the GDD estimates to be able to combine them with the other input parameters for that year. The new estimates of energy intake are referenced (ref 39) and described at several points in the manuscript. Following your comments, we clarified both points. Please see our response to your previous comment for details.

o L420: the goal or rationale for developing this new proxy, or how it is being done, is not clear.

As noted in that section, we developed this proxy for comparison and use in structured sensitivity analyses. In addition, we explain our motivation in the discussion section as follows:

> "One way of incorporating such information [information missing in one of the input datasets] is by developing additional proxies based on combining aspects of availability-based and survey-based estimates, and by using a larger set of proxies in structured sensitivity analyses. We included an energy-corrected proxy based on dietary surveys in our database to facilitate this, and we also developed a combined proxy in which we used survey-based estimates for foods that could be from subsistence production in low-income and lower middle-income countries (Table S11)."

Following your comment, we clarified the paragraph you referred to as follows (after also expanding on the comparison to dietary surveys in the method's previous paragraph):

> "For structured sensitivity analysis, we also developed two additional proxies of food intake. In the first, we used dietary surveys for informing food composition wherever possible (i.e., for the food groups covered by the GDD, Table S9), instead of inferring those from waste-adjusted food availability data. The process of construction followed the same steps outlined in the previous paragraph for converting the reporting to primary commodity equivalents, gap-filling missing foods groups, and disaggregating general food groups to a more detailed set of commodities. To ensure biological plausibility, we also aligned the uncertainty intervals of food intake to the uncertainty levels of total energy intake that are in line with biophysical requirements at current levels of body weight, height, and physical activity levels[39]."

In the method's previous paragraph, we describe the referred-to processing steps as follows:

> "We compared our estimates of food intake to estimates based on dietary surveys. For that, we used the estimates for general food groups contained in the GDD[32]. For comparability, we normalised energy intake to the same estimates of energy intake that we used for our main estimates[39], and to match the reporting in primary commodity equivalents that is used in our main estimates, we calculated milk equivalents based on the calorie contents of dairy products and the calorie density of milk[17], and we converted legumes from cooked to dry equivalents based on ratios derived from the USDA's nutrition database (FoodData Central). As not all food groups were represented in the GDD, we supplemented them with estimates from our main proxy for missing foods (poultry, sugar, soybeans, vegetable oils, palm and coconut oils, fish oil, butter, cream, poultry, other meat, animal fats, stimulants, spices, alcohol, and foods not otherwise classified), and also used our main proxy (in particular, the distribution across specific foods) to disaggregate general food groups into more distinct foods to match the commodity detail of our main estimate (Table S11)."

o L432: what do the authors mean by combining proxies? What does this refer to? Why is it being done?

This refers to the same section in the discussion that we cited in the response to your previous comment:

> "One way of incorporating such information [information missing in one of the input datasets] is by developing additional proxies based on combining aspects of availability-based and survey-based estimates, and by using a larger set of proxies in structured sensitivity analyses. We included an energy-corrected proxy based on dietary surveys in our database to facilitate this, and we also developed a combined proxy in which we used survey-based estimates for foods that could be from subsistence production in low-income and lower middle-income countries (Table S11)."

We hope this is clearer now. Following your comment, we corrected the somewhat odd grammar in this sentence to:

> "For illustrating how different proxies can be combined".

o L472-475: what the authors describe here about adjusting the LCAs is not clear – what specifically is being done to augment/standardize the LCA data, and why? Similarly, L476: "FAO data on food production and yields were used to scale and regionalise estimates" – which estimates are being scaled, and how are FAO data being used to do this?

LCAs often have very different system boundaries. To conduct a consistent meta-analysis, these have to be harmonised, which is what is described there. The regionalisation relates to the LCA estimates and describes how the global results of the meta-analyses were adapted to the regional level. Lastly, the scaling denotes how the footprints were normalised to sum up to national totals as reported by official statistics. The harmonisation, regionalisation, and scaling were done by the authors of the LCA database (Poore and colleagues) and described in detail in the referenced papers. We described these steps here, so that the reader understands where the data are taken from and how those data were derived.

Following your comment, we clarified that the scaling related to matching national statistics and again added the reference to the paper in which the footprints were derived (which was referenced in the previous sentence in that paragraph). The relevant section now reads:

> "The LCAs were standardised by harmonising system boundaries (from inputs to retail) and gap-filling missing steps along the supply chain, which made use of auxiliary estimates (e.g., of post-farm processes) and dedicated process-based models (e.g., of nitrate leaching)[63]. FAO data on food production and yields were used to scale to official statistics and regionalise the estimates[17]"

Comment 1-7:
Results:
- The first indicator described is servings per day of food groups. This is relatively unintuitive (e.g. the authors do not define what a serving of vegetable oil vs. a serving of grains is). I would either remove this and stick to the more intuitive metrics of g/day or kcal/day of food groups, or move the servings/day results to the end of the section.

Reply to Comment 1-7:
Thank you for the comment. We also include your preferred metrics of grams and kilocalories per day. We included servings per day because this is the preferred metric in

most national dietary guidelines. The rationale for this is that most people have a better understanding of how many servings they eat compared to how many grams or calories they eat, granted that each of those are subject to uncertainty. To make our estimates relevant for the public-health community, we would therefore like to keep this additional metric. How many grams are contained in a serving is defined in Table S4 that we refer to in the title of Table 1:

> "Serving sizes are listed in Table S4."

Following your comment, we added an additional reference to Table S4 in the main text at the first mention of servings.

Comment 1-8:
- What do the ranges mean when referring to an "average of 7.5-8.0 servings per person", or "25-30% less roots and vegetables"?

Reply to Comment 1-8:
The ranges refer to the uncertainty intervals. Following your comment, we now clarified this in the text:

> "(with the range referring to the uncertainty of our estimates)"

Comment 1-9:
- Many instances of unclear phrasing:
o Line 119: "changes in food intake were in line with changes in energy intake". Do the authors mean "differences in food intake…"?
o L127: "total energy intake ranged from 7% lower than average in low-income countries…". What is 'average' here? The global average?
o L158: "at the global level, the estimates overlapped with estimates based on dietary surveys for most food groups". Authors need to specify what metric or indicator they are referring to. What does 'overlapped' mean? Are ranges of an indicator overlapping?
o L199: "the survey-based estimates were lower for cropland use". Which two data sources are being compared? What level of diet is being compared here (a global average diet?)
o L202: "the GDD-IA and FBS-based estimates are consistent with official statistics on pasture land" – which country/scale is being compared here in the GDD-IA and FBS? For total global dietary intake?
o L210: "in a dietary cost assessment based on market prices of food, the estimated cost of diet was similar …" – in which country/year, etc?

Reply to Comment 1-9:
Thank you for these questions and suggestions. We addressed each one in turn:

o Line 119: "changes in food intake were in line with changes in energy intake". Do the authors mean "differences in food intake…"?

Yes, thank you. Following your comment, we changed the sentence to:

> "Across socio-demographic groups, differences in food intake were in line with changes in energy intake for most foods (Figure 2)."

o L127: "total energy intake ranged from 7% lower than average in low-income countries…". What is 'average' here? The global average?

Yes, thank you. The average was mention in the previous paragraph ("Compared to the population average"). Following your comment, we inserted it here one more time:

> "Compared to the global population average"

We also inserted "global" at the previous occurrence for clarification.

o L158: "at the global level, the estimates overlapped with estimates based on dietary surveys for most food groups". Authors need to specify what metric or indicator they are referring to. What does 'overlapped' mean? Are ranges of an indicator overlapping?

Thank you for highlighting this sentence. You are right that we should have been clearer that we meant the uncertainty ranges of the new estimates overlap with those of dietary surveys. Following your comment, we now clarified that:

> "At the global level, the uncertainty intervals of the new estimates of food intake overlapped with the uncertainty intervals of dietary-survey estimates for most food groups (7 out of 10) [..]."

Comment 1-10:
Discussion:
- A major limitation of this database, because of the standardization to energy intake requirements, is that absolute intake cannot be measured, nor can absolute environmental footprints, dietary costs, etc, be calculated. Over or underconsumption of macronutrients, etc, can also not be assessed. This should be made more clear.

Reply to Comment 1-10:
There seems to be a misunderstanding. The normalisation to independent estimates of total energy intake was done so that the estimates reflect absolute intake. As explained in some detail in the introduction, neither food-availability estimates nor dietary surveys reflect actual intake: food-availability estimates because they include food waste and dietary-survey estimates because they are subject to substantial misreporting, and global databases such as the GDD normalised intake to one value per broad age group for all countries. The estimates of energy intake we used for our normalisation reflect those levels of intake that are needed to sustain measured levels of body weight, height, and physical activity, which therefore is a more objective measure of actual intake than dietary surveys or national food balances of production and trade. We thought we were very explicit about this, both in the introduction and in the discussion section. For example, in the introduction we state:

> "Here we develop a new global dietary database that is particularly designed for use in impact assessments that require (i) coverage of complete diets, (ii) absolute intake levels that minimise risk of over or underestimation, and (iii) granular socio-

demographic detail. Examples of such impact assessments include analyses of nutritional adequacy, dietary risk assessments, environmental analyses of diets[34,35], estimates of the affordability of diets[36], and assessments of the proportion of undernourishment[37,38]. For constructing the Global Dietary Database for Impact Assessments (GDD-IA), we combined the key strengths of existing data and used newly estimated data to address existing shortcomings. To derive the composition and overall scale of dietary intake, we used estimates of food available in a country[17] and first subtracted estimates of food waste at retail and household levels[20] and then normalised intake to estimates of total energy intake that are based on anthropometric measures of body weights, heights, and physical activity[39]. This normalisation procedure ensured that our estimates are biophysically plausible."

And in the discussion section, we re-iterate and discuss this as follows:

> "Dietary intake has major implications for health, the environment, and the economy. However, current global estimates of food intake are fundamentally uncertain and often not regionally comparable. We developed a new Global Dietary Database for Impact Assessments (GDD-IA) that combines the strengths of existing data sources. For deriving food intake at the national level, we used regionally comparable statistics on food available for human consumption[17] from which we subtracted waste at retail and household levels[20] and then normalised to estimates of total energy intake derived from measurements of body height, weight, and physical activity[39]. For deriving comparable socio-demographic trends across age groups, sex, and urban/rural residence, we used estimates from dietary surveys[32] and again normalised those to the estimated energy intake of the relevant population group[39]. By construction, our estimates are in line with observed trends in anthropometric measures such as body mass index[40,41], they include both processed and unprocessed foods[17,32], and they cover complete diets at global, regional, national, and socio-demographic levels.
>
> The GDD-IA addresses the main shortcomings of existing estimates. [..] Lastly, normalising to independent estimates of total energy intake ensures that estimated intake is biologically plausible [..]. We therefore think our estimates can inform and be used for more biologically grounded assessments of both food intake[..]."

Following your comment, we clarified the last sentence of the quoted paragraph in the introduction to read:

> "This normalisation procedure ensured that our estimates are biophysically plausible and represent absolute levels of intake."

Comment 1-11:
- L240: the GDD-IA addresses the main shortcoming of existing estimates that attempt to be globally comparable (not simply existing dietary estimates … as nationally-representative dietary surveys on their own may be more advantageous than using the GDD-IA as they would allow for estimates of absolute intake). Similarly for L242, national-level policy planning could in many cases be better served by using the national dietary survey, rather

than the GDD-IA estimated intake for the same country.

Reply to Comment 1-11:
We actually do think that our database addresses the shortcomings of existing dietary estimates in general. As explained above and in the manuscript, our new estimates do reflect absolute intake, whereas dietary surveys do not because they are known to be subject to substantial misreporting, also when used individually. In addition, global databases adjust estimates to rather arbitrary levels of energy intake that are kept constant across countries, but such adjustments are also often made when compiling national-level surveys. We discuss the shortcomings of dietary surveys in the introduction as follows:

> "Another source of data are dietary surveys, including 24-hour recalls and food-frequency questionnaires. They provide a rich source of information, including on socio-demographic characteristics, and are considered the gold standard in public health research[15]. However, they are prone to misreporting, e.g., due to imprecision of communicating and reporting serving sizes[24], difficulties in the recall of intake[15], and tendencies in responders to report what is perceived as socially desirable[25,26]. As a result, dietary surveys are known to substantially underestimate total calorie intake[27–31]. Datasets such as the Global Dietary Database (GDD) have standardised and harmonised individual-level dietary surveys, but they normalised food intake to the same total energy intake in every country and most socio-demographic groups, and therefore do not capture actual food intake[32,33]. In addition, the GDD focussed on a selection of food groups and therefore does not capture complete diets, which makes the estimates unsuitable for assessments for which overall intake matters, including food security, affordability, nutritional adequacy, and environmental impacts."

Following your comment, we clarified in the methods section that most of the surveys used in the GDD were nationally representative:

> "The [GDD] estimates are based on data from about 1220 dietary surveys, most of which were nationally representative, covered all age groups, and used food-frequency questionnaires or 24-hour recalls[32]."

Comment 1-12:
- L257-260: it is unclear what the authors are referring to, or what point is being made. What are "estimates of remaining foods from our primary proxy"?

Reply to Comment 1-12:
We explained in the previous sentence in that paragraph and earlier in the manuscript that the global database of dietary surveys does not contain all food groups that make up complete diets. For deriving total food intake, one therefore has to supplement missing foods (e.g., poultry and oils) from other datasets. This is what is meant with "remaining foods". Following your comment, we changed "remaining foods" to "missing foods":

> "An alternative to deriving food intake from statistics on food availability could have been to adopt estimated intake from dietary surveys, at least for the foods reported in global databases such as the GDD. However, the variations in survey-based

estimates were so wide that they resulted in biophysically implausible intake when combined with estimates of missing foods from our primary proxy"

In addition, we clarified the section in the results that the discussion is referring to as follows:

> "The large range of uncertainty intervals resulted in a range of total energy intake of 1620 to 3615 kcal/d when gap-filled with estimates from our main proxy for foods not represented in the dataset of survey estimates (i.e., of foods that make up complete diets but are not represented in the GDD such as poultry and oils, see Methods and Table S10)."

Comment 1-13:
- L289: what do authors mean by "additional proxies". Are these other iterations of the GDD-IA intake estimates, that use different adjustment methods?

Reply to Comment 1-13:
A proxy is a set of estimates for something, in this case food intake. We use the word "proxy" to stress that estimated intake is fundamentally uncertain. Additional proxies denote different sets of estimates of food intake. The GDD-IA contains one primary one that is described in the main results as primary proxy. As explained in the paragraph you are referring to, we also provide a set of additional estimates that can be used in structured sensitivity analysis: one which uses as much data from dietary surveys as possible, and one which uses dietary-survey data for foods that might be produced from subsistence production. Our primary estimates reflect our best guess based on the three sources of available data (dietary surveys, food balance sheets, estimates of energy intake) and what we think are reasonable assumptions, but we think it would be good practice to conduct sensitivity analyses with proxies of dietary intake that are based on other assumptions, and the two additional proxies illustrate this point. We hope the referenced table (Table S11 in the original manuscript) also clarifies this point:

> "We included an energy-corrected proxy based on dietary surveys in our database to facilitate this (Methods), and we also developed a combined proxy in which we used survey-based estimates for foods that could be from subsistence production in low-income and lower middle-income countries (Table S11)."

In the methods section, we describe the additional proxies as follows:

> "For structured sensitivity analysis, we also developed two additional proxies of food intake. In the first, we used dietary surveys for informing food composition wherever possible (i.e., for the food groups covered by the GDD, Table S9), instead of inferring those from waste-adjusted food availability data. [..]
>
> For illustrating how different proxies can be combined, we also developed one proxy in which we supplemented the main FBS-based proxy with GDD-based estimates for foods with a high proportion of subsistence production. [..]"

Comment 1-14:
- L291: this description is unclear – what is an energy-corrected proxy based on dietary

surveys, and what does it facilitate?

Reply to Comment 1-14:
As noted in our paragraph on dietary surveys (and the summary in the discussion section), dietary surveys do not reflect actual energy intake. For pairing the socio-demographic information from dietary surveys with waste-adjusted food availability data, we first energy-corrected both datasets to independent estimates of energy intake. This is what we mean with "energy corrected". We also explained this in detail in the dedicated section in the methods ("Developing additional proxies of food intake", some of which we quoted in our previous responses). Following your comment and to better guide the reader, we now refer to this section from the main text (please see the quoted text in our previous response).

Comment 1-15:
- L304-306: what about data from 2000-2010? Was this harmonized?

Reply to Comment 1-15:
Thank you, this was a typo. This was meant to read "1990 to 2010", and it was harmonised as well. As stated in the sentence you refer to, the harmonisation relates to calorie densities ("harmonised [..] by aligning calorie densities") which differed in earlier estimates (1990 to 2010) due to a different method of construction. We detail this more in the methods section as follows:

> "Estimates prior to 2010 are based on a different method for deriving food balances. To avoid discontinuities, we harmonised the calorie ratios between the two datasets and re-calculated intake in grams prior to 2010 (Supplementary Information, section SI1), in line recommended practice[44]."

Following your comment, we replaced "calorie ratios" with "calorie densities" for consistency between the discussion and the methods, and we inserted a reference to the methods section to better guide the reader. The relevant section in the discussion now reads:

> "For our analysis, we harmonised internally consistent estimates of food balances for the years 2010 to 2020 (released up to 2023) with earlier estimates for the years 1990 to 2010 by aligning calorie densities (Methods)"

Comment 1-16:
- L314: did the authors apply the new estimates of food waste to compare with body weight trends, or is this a citing finds from another paper?

Reply to Comment 1-16:
We cited the new estimates of food waste in this sentence (ref 43 in the initial manuscript). The reference contains a supplementary table which details estimated levels of food intake (final food supply net of waste) that are based on their estimates. We compared those levels to estimates of body weight across countries and found them to be inconsistent. For example (and there are many more), they reported estimates of net food supply in 2014 that were larger for many East Asian countries (e.g., Thailand, Malaysia, Cambodia) than for the USA, in contrast to the USA having a much larger prevalence of overweight and obesity and, related to that, much larger energy requirements (also after accounting for differences in

physical activity). We communicated these inconsistencies (which curiously are not present in the older Gustavsson estimates) to the authors, and they agreed with us that they present a problem. We still think the study by Gatto and Chepeliev presents a much-needed initiative for updating waste fractions, so we did not want to overly criticise their study in our resource paper. Instead, we hope to be able to use updated estimates in the future and therefore decided to focus on that in our discussion.

Response to Reviewer #2

Comment 2-0:
This manuscript presents a significant and timely advancement in global dietary data. The development of the Global Dietary Database for Impact Assessments (GDD-IA) fills an important methodological gap, and the integration of FAO Food Balance Sheets (FBS) with anthropometric energy-requirement estimates represents a notable innovation. The following comments aim to strengthen clarity, justification, and transparency.

Reply to Comment 2-0:
Thanks a lot for your comments and suggestions. Please find our point-by-point response below.

Comment 2-1:
1. Clarify the triangulation strategy
The manuscript notes that dietary surveys such as 24-hour recalls and FFQs “are considered the gold standard in public health research” (lines 47–48), yet immediately highlights substantial misreporting challenges—including serving-size misestimation, recall difficulties, social desirability bias, and systematic under-reporting of energy intake (lines 49–53). This presents a conceptual inconsistency. If surveys have such well-documented biases, it requires clarification in what sense they serve as a gold standard, and for which aspects of dietary intake this designation applies (absolute levels, food-category composition, or demographic gradients).

This issue carries particular importance because GDD-IA relies on survey-derived relative intake patterns to disaggregate FBS-based national totals across sex, age, and residence. The authors should clarify why these relative differences are assumed to be reliable even when absolute survey intake is not, and whether empirical evidence supports the idea that misreporting biases are proportional—or at least stable—across demographic groups.

More broadly, the manuscript would benefit from discussing existing comparisons between FBS data and high-quality national dietary intake surveys, and explaining why discrepancies are interpreted primarily as survey underestimation rather than potential FBS overestimation. Identifying examples where FBS-normalized intake aligns—or diverges—from rigorous national surveys would help readers understand where GDD-IA is likely to perform well and where uncertainties remain.

Reply to Comment 2-1:
Thank you for the comment. Public health research often relies on data collected through surveys, especially for behavioural aspects that don’t lend themselves to be assessed by objectives measures. For that reason, assessing individual-level dietary intake through dietary surveys has a long history, and a large body of literature has tried to improve survey methods and design, whilst also pointing out the now well-known caveats. For example, 24-hour recalls are considered more accurate than food-frequency questionnaires, but they are also more resource-intensive and therefore not always suitable for large-scale national surveys. So it is not a conceptual inconsistency that a general method is considered the gold standard in a specific domain, whilst at the same being subject to substantial shortcomings.

We highlighted what would resolve the conceptual problems of measuring food intake in the discussion section as follows:

> "Ideally, we would have also preferred to use more direct measurements of food intake, but the degree of inconsistencies in dietary-survey estimates made them unsuitable for constructing a consistent and regionally comparable database of food intake that can be used in impact assessments at the population level. Efforts to measure food intake more directly include the use of image-assisted dietary assessments which can improve the misreporting of serving sizes and incomplete recall[46,47], and of dietary or recovery biomarkers which can be used to validate the accuracy and plausibility of reported intake[31,48]. Before more direct and objective estimates of food intake become widely available, critically evaluating and triangulating exiting estimates, as we have done in this study, is one way of addressing existing shortcomings and improving estimates of dietary intake and related assessments."

You are right in pointing out that the disaggregation of national level intake by socio-demographic groups is subject to the caveats that generally are related to dietary surveys. Unfortunately, there is no other regionally comparable data that would allow us to disaggregate the estimates in this way. To nevertheless make the estimates more reliable, we energy-corrected the dietary-survey estimates before using them to derive socio-demographic variations in intake. By doing so, we made sure that the overall level of food intake by each socio-demographic group is biophysically plausible, which addresses the survey-related issues of general over and underreporting. We explained this approach in the introduction, methods, and discussion sections. For example, in the introduction we note:

> "In addition, we used estimates from dietary surveys to derive the degree of variation in intake across sociodemographic groups and for estimating the intake of processed foods. For the former, we mapped the variation in intake across sex, age groups, and urban/rural residence inferred from dietary surveys[32] to the food-group detail of the national estimates, and normalised them before and after the mapping to the estimated energy intake in those population groups[39]. As before, the normalisation ensures the estimates are biophysically plausible, whereas the mapping downscales the survey estimates from general food groups (e.g., grains) to specific commodities (e.g., wheat)."

We think our triangulation method (using both FBS and survey data, normalised to external estimates of overall food/energy intake) addresses the major caveats that apply when FBS or survey data are used on their own. However, we also acknowledge that the original caveats also apply to our new estimates albeit to a lesser degree. We note this in the discussion section as follows:

> "In addition to the various improvements over existing estimates, our new estimates of food intake are also subject to several caveats. Many of them carry over from those associated with the data sources used in their construction, including estimates of food availability, food waste, estimated energy intake, and dietary surveys. [..]"

You are right to note that the differences between survey-based and FBS-based estimates could be due to either data source. As we note in the introduction and discussion sections, "food-availability estimates have been found to overestimate actual intake if not carefully adjusted for food waste[18,19], and estimates of food waste are rarely updated[20]", whereas "dietary surveys are known to substantially underestimate total calorie intake[27–31]." The references we provide contain detailed comparisons and assessments of each data source. For example, Del Gobbo and colleagues (reference 18 in the original manuscript) compared survey estimates with food availability data, finding that the latter overestimates compared to the former. We could have discussed this paper in more detail, but did not think this would be necessary, because (a) the survey data they used is known to be subject to underreporting, and (b) the food availability data they used includes the amount of food that is wasted before being consumed and therefore is not considered an actual proxy for food intake. We consider it as a given that FBS data overestimates actual intake if not adjusted for food waste (in contrast to waste-adjusted availability data which we use as a one data source for our estimates).

For us, it was more instructive to compare the results of our new proxy (based on energy-corrected and waste-adjusted FBS estimates at the national level) to dietary-survey estimates, and to identify differences and likely explanations. We were careful not to assign value judgements regarding which data over or underestimates in the main results section, and only provided interpretation of the deviations in the discussion section. In line with your comment, we provided reasoning for deviations in either source. When the deviations were in line with known caveats of dietary surveys, we interpreted them in this way, and when the deviations were in line with known caveats of FBS data, we also interpreted them in this way, and if possible sought compounding evidence. For example, the potential overestimate in surveys of red-meat intake in middle-income countries is compounded by exceeding food availability estimates (that include waste), and by the associated land use exceeding official statistics. In contrast, the potential underestimate in our proxy of foods that might be produced by subsistence agriculture not recorded in FBS statistics is compounded by waste-adjusted energy intake being below estimates of energy intake that are based on anthropometric measures (Table S6 and ref 39).

Following your comment, we added the compounding information mentioned in the latter case to the relevant part in the discussion section (the discussion of the other case already included the compounding information). The relevant section now reads:

> "The comparison also highlighted aspects where our primary proxy could be improved. In particular, the much larger survey-based intake of vegetables, fish, and eggs in lower middle-income and low-income countries could be due to a proportion of those foods being produced for own consumption by small-holder farmers or subsistence agriculture, which are not being recorded in food availability statistics (see, e.g., Table S6)[39]."

Comment 2-2:

2. Provide more detail on how survey-derived demographic scaling works

The demographic scaling procedure is central to GDD-IA, but several steps need additional clarification. The manuscript states that survey-derived relative differences across sex, age, education, and urban/rural residence are applied to disaggregate national totals (lines 100–

109). However, given the acknowledged misreporting in dietary surveys (lines 49–53), it is unclear why these demographic ratios should be considered reliable indicators. This concern is particularly relevant for the urban–rural distinction, where survey representativeness and data quality vary widely and misreporting patterns may differ.

Furthermore, many countries have multiple surveys that differ in methodology (24-hour recall, FFQ, HCES) and survey year. The manuscript should clarify how demographic ratios are derived when multiple surveys exist, whether ratios differ across survey types, and how within-country heterogeneity is handled.

It is also unclear how demographic ratios are applied to food groups that are poorly measured or entirely absent in surveys, given that the surveys cover only 14 food groups and 7 beverages while GDD-IA includes 37 food groups. The authors should also explain how demographic scaling is handled in countries without any dietary surveys, as this is essential for understanding global coverage and comparability.

Finally, the manuscript mentions that intake is normalized both before and after applying demographic ratios, but the rationale for this double normalization—and its quantitative effect on the resulting demographic patterns—is not explained. A brief example or schematic would substantially improve transparency.

Reply to Comment 2-2:
Thanks for the comment. You are right to note that the socio-demographic ratios are subject to uncertainty. As mentioned in our response to your previous comment, we addressed some of the uncertainty by energy-correcting the survey estimates from the GDD before using them to derive the coefficients of socio-demographic variation. We explain this in the introduction as follows:

> "In addition, we used estimates from dietary surveys to derive the degree of variation in intake across sociodemographic groups and for estimating the intake of processed foods. For the former, we mapped the variation in intake across sex, age groups, and urban/rural residence inferred from dietary surveys[32] to the food-group detail of the national estimates, and normalised them before and after the mapping to the estimated energy intake in those population groups[39]. As before, the normalisation ensures the estimates are biophysically plausible, whereas the mapping downscales the survey estimates from general food groups (e.g., grains) to specific commodities (e.g., wheat)."

And detail it further in the methods section:

> "For comparability, we processed the GDD data in several ways. First, we projected the GDD estimates from 2018 to 2020 based on trends in food availability, a method in line with their framework for gap-filling estimates[32]. Second, we used the new estimates of energy intake[39] (per population group in each country) to re-normalise the GDD estimates to those levels (Tables S5-S6). Third, we calculated ratios of food intake across the socio-demographic groups and for the food groups covered by the GDD (Table S8). Fourth, we mapped those ratios to the food groups covered in our

database (Table S9) and normalised to the estimated energy intakes one more time to ensure that the mapping between food groups preserved estimated intake."

As mentioned there, the mapping is detailed in Table S9 and the second normalization was done to ensure that the mapping of food groups did not introduce any inconsistencies in overall food intake. Those could occur because the absolute intake levels for specific food groups in the GDD differ from the absolute levels of intake in the waste-adjusted FBS data, also after each data source is energy-corrected to the same overall level of energy/food intake. For example, if red-meat intake was greater in the energy-corrected GDD estimates but lower in the energy-corrected FBS-based estimated, then the socio-demographic variation mapped from the former to the latter covered less energy intake, with the remainder of total energy intake covered by other food groups with different coefficients of socio-demographic variation. In short, the distribution of total energy intake across food groups differs between the GDD-based estimates and the FBS-based ones, and this is what requires a second normalisation as otherwise the sum of energy from each food group could be misaligned with the estimates of total intake.

Following your comment, we added this more comprehensive explanation to the relevant section in the supplementary information ("SI4. Variation across socio-demographic groups") and now refer to it from the main methods section which we also clarified as follows:

> "We processed the GDD data in several ways to be able to consistently combine them with our national-level estimates of food intake. To match our time series, we projected the GDD estimates from 2018 to 2020 based on trends in food availability, a method in line with their framework for gap-filling estimates[32]. To align the GDD estimates with biophysically grounded estimates of energy intake, we used the estimates of energy intake that are in line with current biophysical requirements per population group in each country[39] to re-normalise the GDD estimates to those levels (Tables S5-S6). We then calculated ratios of food intake across the socio-demographic groups and for the food groups covered by the GDD (Table S8) and mapped those ratios to the food groups covered in our database (Table S9). To ensure that the mapping between food groups and source data preserved the overall levels of energy intake, we also normalised the final estimates for each socio-demographic group to their estimated energy intakes. Section SI4 provides additional descriptions and an overview of the data used in these steps."

Regarding your question on the methodology used for the surveys and potential data gaps: we did not process any survey data for this study but used the survey estimates contained in the GDD. Ref 32 in our initial manuscript contains a detailed description of the GDD. It is based on standardised data from about 1220 surveys, of which 41% were from food-frequency questionnaires, 23% from 24-hour recalls, 16% from demographic-health-survey questionnaires, and the remainder from biomarkers and household surveys. Imputation modelling was used to account for differences in survey design, representativeness, dietary assessment methods and dietary factor definitions to estimate normalised dietary intake by age, sex, and urban/rural residence.

Following your comment, we added the additional details to our main methods section, which now states:

> “We disaggregated national food intake by age group, sex, and urban/rural residence based on trends in intake inferred from dietary surveys and normalised to estimated energy intake per population group. We sourced the differences in dietary intake by sociodemographic group from the GDD[32]. The database contains estimates of dietary intake of 14 major food groups (as well as beverages and nutrients) and stratified by age (in five-year groups), sex (female, male), and residence (urban, rural) for 184 countries and the years 1990-2018. The estimates are based on data from about 1220 dietary surveys, most of which were nationally representative, covered all age groups, and used food-frequency questionnaires or 24-hour recalls[32]. Estimated intake in the GDD was normalised to a constant level of energy intake per age group (2000 kcal/d for ages 11-74, 1700 kcal/d for ages 75+ and 6-10, and 700-1300 kcal/d for ages 5 and below).”

Regarding your question on coverage and data gaps: the data sources used already had global coverage, so we did not need to conduct any gap-filling. Instead, when one of the three data sources did not have data for a specific country, we then omitted this country in our final estimates. Following your comment, we compiled a table of countries covered by the different data sources and our final estimates (new Table S10). We added an explanatory note in the methods section in which we refer to the new table as follows:

> “The three datasets used to the construct the GDD-IA (FBS data, GDD data, and estimates of energy intake) have global coverage. When a country was not represented in one of the datasets, we dropped that country from our final estimates. This resulted in estimates for 171 countries (Table S10).”

Comment 2-3:
3. Address subsistence production and informal food sources more directly
Subsistence production, informal food acquisition, and home-produced foods play important roles in many low-income settings, yet they are not fully captured in FBS data. Although the manuscript acknowledges this limitation and proposes an alternative “subsistence-adjusted proxy” for vegetables and fish in low-income and lower-middle-income countries, the adjustment appears narrow and somewhat ad hoc.

It would strengthen the manuscript to more clearly quantify which food groups and regions are most affected by subsistence production, the likely magnitude of underestimation, and whether household consumption and expenditure survey data could be used to calibrate or cross-validate these gaps. Because national totals affect all downstream demographic estimates, more explicit characterization of this uncertainty is important for interpreting GDD-IA, especially in low-income contexts.

Reply to Comment 2-3:
We were discussing for some time how to systematically address subsistence production in our new proxy. Unfortunately, there are no regionally comparable estimates of the types and quantities of foods that are produced in subsistence agriculture. In absence of this, we decided to not introduce any ad-hoc assumption to our main proxy and instead focussed on energy-correcting the dietary composition we have data for. By doing so, the main proxy accounts for the calories obtained from subsistence production because they do show up in

the external estimates of energy intake (as those are based on the energy intake required to sustain measured levels of body weight, height, and physical activity). Table S6, which shows the ratio of estimated energy intake between anthropometric measures and waste-adjusted FBS estimates, indicates that actual energy intake was 13% greater in 2020 than what is covered by waste-adjusted FBS data, something that is corrected by applying those ratios. The energy correction applies to all food groups and therefore implicitly assumed that a proportion of all foods are from subsistence production.

We included an additional proxy in our dataset to show how information on subsistence production could be incorporated in our new estimates of food intake. For this, we interpreted the deviation in the intake of specific food groups in lower-income countries between survey-based estimates and our main proxy as suggestive of subsistence production, focussing on positive deviations that were statistically significant (e.g., vegetables and fish), and then used the survey-based estimates for the final proxy (before energy correcting the estimates one more time). We think this is better than making ad-hoc assumptions, and it hopefully illustrates how such information can be incorporated in the estimates. However, because several additional steps and assumption were required, we did not want to use this proxy as main estimates, but instead offer this in addition. We describe the construction of the additional proxy in the methods section as follows:

> “For illustrating how different proxies can be combined, we also developed one proxy in which we supplemented the main FBS-based proxy with GDD-based estimates for foods with a high proportion of subsistence production. Based on our comparison between the main proxy and survey estimates, we chose vegetables and fish in low-income and lower middle-income countries as targets for adjustment. The subsistence-adjusted proxy was constructed as follows. First, intake values for the targeted foods in the specific regions were adapted from the survey-based proxy. Second, intake values for the remaining foods and regions were adapted from the main proxy. Third, overall energy intake was calculated and normalised to estimated levels for consistency. Table S11 provides an overview of the additional proxies.”

Following your comment, we clarified in the discussion section that also the main proxy accounts for the energy intake that might be from subsistence production:

> “In comparison, our main estimates implicitly account for subsistence production by energy correcting the waste-adjusted food availability data, but this implicitly assumes that a proportion of all foods comes from subsistence production.”

Comment 2-4:
4. Reconsider the inclusion of the death-attribution, environmental, and cost demonstrations The environmental and cost analyses appear intended to illustrate the utility of GDD-IA, but the current demonstrations raise methodological questions and are not as strong as the core contribution. Because GDD-IA and FBS share the same national-level intake totals, their environmental and cost outputs inevitably appear very similar, which limits the interpretive value of the comparisons. In addition, the price data used (retail food prices from the ICP) show considerable within-group variation, making food-group-level cost comparisons difficult to interpret. Similarly, the differences in dietary risk–attributable deaths across GDD-IA, GDD, and FBS appear driven largely by whole-grain intake differences, but this is not fully

explained.

For these reasons, the manuscript may be strengthened by removing Figure 5 or simplifying it substantially, accompanied by a concise statement noting that GDD-IA can support downstream applications without presenting detailed examples that are difficult to interpret and not central to the methodological advance.

Reply to Comment 2-4:
Thanks for the comment. Initially, we developed the manuscript without this section, but were then asked by the editors to illustrate how our new estimates differ from existing ones when applied. We agreed to the merit of such a comparison and aimed to focus on what differs between the estimated impacts. Sometimes the differences were due to not having estimates for certain food groups in the existing datasets (e.g., whole grains and processed meat in FBS-based proxies, which affects the health assessment; or an absence of some foods in the GDD, which affects the cost assessments). Other times, the differences were due to differences in the absolute levels of intake (e.g., lower estimates of fruits and vegetables in the GDD, which affects the health assessment; and higher estimates of red meat intake which affects the environmental assessment). We also highlight where estimates are relatively similar (e.g., in the environmental assessment between the FBS-based estimates and our new ones).

We think these illustrations do capture the qualitative differences between the new estimates and existing ones when applied in impact assessments (despite within-group variation of some impact parameters such as prices, which however apply similarly to the different datasets), so we would like to keep them in the manuscript. We do stress that these applications are not for providing detailed assessments on their own, but are only for illustrating the differences between the datasets. For the former, we agree with you that are more detailed treatment and discussion would be necessary. We phrased this in the main results section as follows:

> “We conducted a series of impact assessments (see *Methods*) to illustrate the differences between the new database (GDD-IA), a global database of dietary surveys (GDD), and waste-adjusted food availability data (FBS).”

And in the methods section, we note:

> “We conducted a series of dietary impact assessment to illustrate the use of the new database of intake estimates, and to compare the associated results to those based on other proxies for intake.”

Following your comment, we qualified the illustrations to relate to the “relative” differences (in the results) and the “relative” use (in the methods) for additional emphasis on the illustrative nature of these applications.

<u>Response to Reviewer #3</u>

Comment 3-1:
INTRODUCTION
The authors have done a great job in summarizing the problem statement, the positioning of this study and its contribution to the literature. I do not have specific remarks on the introduction.

Reply to Comment 3-1:
Thank you, also for your other comments and suggestions. We address them in our one-by-one response below.

Comment 3-2:
RESULTS
The results section is well structured and provides an overview of the constructed database. To enrich its message, I would advise the authors to integrate an additional short paragraph where country- or region-specific estimates of total food intakes are reported and described, illustrating the variation in total calorie intakes. Something you have now in Figure 1b but that is missing in the main text.

Reply to Comment 3-2:
Thanks for the comment. We describe the variation in total food/energy intake in the results section as follows:

> "Compared to the population average, energy intake was 40% lower in children, 5% higher in adolescents, 15% higher in young adults, 10% higher in middle-aged adults, and 5% lower in older adults. Across sexes, it was 20% lower in women compared to men, and across residence, it was 5% lower in rural residences compared to urban residences. The variations differed by region, but with similar trends across socio-demographic groups within each region.
>
> Across regions, diets and energy intake differed substantially (Figure 2). Compared to the global population average, total energy intake ranged from 7% lower than average in low-income countries to 10% greater in high-income countries."

The absolute numbers are presented in Table S5 in the Supplementary Information. Following your comment, we added the absolute value of the global population average to the main text in parenthesis and a reference to Table S5:

> "Compared to the global population average (2160 kilocalories per person per day, kcal/d), energy intake was [..] (Table S5)."

Comment 3-3:
I believe more explanation is needed for Figure 2. The results by gender and residence appear to be specular across the two categories (male-females, rural-urban). Is this by construction of the used variable? Please add a couple of sentences to explain why is that so and comment on the findings.

Reply to Comment 3-3:
The results are symmetric because they express variation from the average. That means that if men generally consume more food than average, women consume less. The same is true for the trends across urban compared to rural residences. Both trends are in line with greater energy intake (i.e., total food intake) in men and by urban populations. In the former, this reflects greater heights in men which are associated with greater energy requirements. In the latter, it reflects greater income in urban populations which in most countries (except high-income ones) is associated with greater food intake, an observation sometimes described as dietary transition.

In the main results section, we described these trends as follows:

> "Across sexes, it [energy intake] was 20% lower in women compared to men, and across residence, it was 5% lower in rural residences compared to urban residences."

Following your comment, we clarified this as:

> "Across sexes, it was 20% lower in women compared to men (mostly reflecting physiological differences), and across residence, it was 5% lower in rural residences compared to urban residences (reflecting income-dependent dietary transitions)."

Comment 3-4:
Lines 163-165: I believe it is necessary to further explain gap-filling procedure in detail. This comment also applies to the methodology or SI section where I think you should expand on the description of such procedure.

Reply to Comment 3-4:
Thanks for the comment. As mentioned in the introduction, the available global database of dietary surveys does not cover all foods that make up diets. We describe this as follows:

> "In addition, the GDD focussed on a selection of food groups and therefore does not capture complete diets, which makes the estimates unsuitable for assessments for which overall intake matters, including food security, affordability, nutritional adequacy, and environmental impacts."

For comparing our new estimates to dietary surveys, we mostly focused on food groups that were present in our data and in the GDD, but in addition we also compared the estimates of total energy intake for which we had to gap-fill the GDD estimates. We explain in the methods and in the supplementary information (Table S10) which foods groups we had to gap-fill and how. In particular, we state:

> "As not all food groups were represented in the GDD, we supplemented them with estimates from our main proxy for missing foods (poultry, sugar, soybeans, vegetable oils, palm and coconut oils, fish oil, butter, cream, poultry, other meat, animal fats, stimulants, spices, alcohol, and foods not otherwise classified)"

These foods were not specifically present in the GDD and also not part of the more general food groups that were represented. With “supplemented” we mean that we simply used the estimates of our new proxy for this.

Following your comment, we clarified the sentence in the results section that mentioned gap-filling to now read:

> “The large range of uncertainty intervals resulted in a range of total energy intake of 1620 to 3615 kcal/d when gap-filled with estimates from our main proxy for foods not represented in the dataset of survey estimates (i.e., of foods that make up complete diets but are not represented in the GDD such as poultry and oils, see Methods and Table S10).”

In addition, we rewrote the relevant section in the methods for clarity, which now reads:

> “We compared our estimates of food intake to estimates based on dietary surveys. For that, we used the estimates for general food groups contained in the GDD[32]. For comparability, we normalised energy intake to the same estimates of energy intake that we used for our main estimates[39], and to match the reporting in primary commodity equivalents that is used in our main estimates, we calculated milk equivalents based on the calorie contents of dairy products and the calorie density of milk[17], and we converted legumes from cooked to dry equivalents based on ratios derived from the USDA’s nutrition database (FoodData Central). As not all food groups were represented in the GDD, we supplemented them with estimates from our main proxy for missing foods (poultry, sugar, soybeans, vegetable oils, palm and coconut oils, fish oil, butter, cream, poultry, other meat, animal fats, stimulants, spices, alcohol, and foods not otherwise classified), and also used our main proxy (in particular, the distribution across specific foods) to disaggregate general food groups into more distinct foods to match the commodity detail of our main estimate (Table S11).”

Comment 3-5:
DISCUSSION
I completely miss a comparison with currently available databases and an explanation of why the provided estimates in this manuscript align or differ with the current literature. This also applies for the amount of country-level waste calculations, which seems to be key in obtaining the final estimates of net food intakes.

Reply to Comment 3-5:
We compare our estimates to available database in several ways. Our main comparison is to estimates from dietary surveys which is detailed in a dedicated section in the results entitled “Comparison to estimated intake from dietary surveys” and which we discuss in three paragraphs in the discussion section:

> “An alternative to deriving food intake from statistics on food availability could have been to adopt estimated intake from dietary surveys, at least for the foods reported in global databases such as the GDD. However, the variations in survey-based estimates were so wide that they resulted in biophysically implausible intake when

combined with estimates of missing foods from our primary proxy, ranging from a population-level energy intake of 1620 kcal/d (a level similar to that of children and adolescents) to 3610 kcal/d (a level similar to that of athletes). An additional inconsistency of the survey-based estimates was that estimates for specific food groups such as red meat exceeded food-availability statistics (with waste included) for upper middle-income countries where statistics on food availability are relatively good. The large differences in the reported intake in red meat resulted in estimated environmental impacts that were inconsistent with independent statistics, e.g., on pasture use, as illustrated in our example applications. Based on these consistency checks, we decided against using dietary surveys for deriving a complete diet proxy, and instead used their variation across sociodemographic groups to supplement our main estimates.

A detailed comparison of our primary proxy to one based on dietary surveys indicated large differences. However, most of the differences can be explained by known shortcomings of dietary surveys which have been found to be prone to misreporting due to a variety of factors, including a tendency of respondents to answer according to social norms (social desirability/approval bias)[25,26], poor understanding and unclear communication of servings sizes[24], and problems with recalling intake from memory[15]. Specifically, the higher estimated intake from surveys of red meat in middle-income countries could be due to red meat being perceived as high-status foods, whereas the lower estimated intake of roots and legumes in low and middle-income countries could be due to these foods being perceived as low-status foods. The lower estimated intake of fruits and vegetables in upper middle-income countries could indicate challenges in understanding and communicating serving sizes, whereas the lower estimated intake of nuts in high-income countries could be due to the use of nuts in processed foods and the consequent challenge in recall.

The comparison also highlighted aspects where our primary proxy could be improved. In particular, the much larger survey-based intake of vegetables, fish, and eggs in lower middle-income and low-income countries could be due to a proportion of those foods being produced for own consumption by small-holder farmers or subsistence agriculture, which are not being recorded in food availability statistics (see, e.g., Table S6)[39]. One way of incorporating such information is by developing additional proxies based on combining aspects of availability-based and survey-based estimates, and by using a larger set of proxies in structured sensitivity analyses. We included an energy-corrected proxy based on dietary surveys in our database to facilitate this (Methods), and we also developed a combined proxy in which we used survey-based estimates for foods that could be from subsistence production in low-income and lower middle-income countries (Table S11). In comparison, our main estimates implicitly account for subsistence production by energy correcting the waste-adjusted food availability data, but this implicitly assumes that a proportion of all foods comes from subsistence production."

Second, we compare our estimates to waste-adjusted availability data, both in general and in the dietary impact assessments. In the discussion section, we discuss the differences as follows:

> “The GDD-IA addresses the main shortcomings of existing estimates. Compared to waste-adjusted food availability data, the GDD-IA includes estimates for socio-demographic groups within countries, which allows for more detailed analyses and policy planning. The GDD-IA also includes estimates for foods that are particularly relevant for health but not included in food availability data such as whole grains and processed meat. This coverage allows for more comprehensive dietary risk assessments, as illustrated in our example applications. Lastly, normalising to independent estimates of total energy intake ensures that estimated intake is biologically plausible, and implicitly controls for uncertainties in and irregular updates of food-waste estimates that affect waste-adjusted food availability data. For example, assuming constant waste fractions as existing impact assessments have done[42] would overestimate intake if wastage rates have increased (Table S6). We therefore think our estimates can inform and be used for more biologically grounded assessments of both food intake and food waste, especially when paired with regular updates of commodity-specific wastage rates[43].”

The referred-to table (Table S6) provides an overview of differences in total energy intake between the independent estimates of energy intake that are in line with biophysical requirements and the energy intake obtained from waste-adjusted food availability data. It indicates the differences between our new estimates and waste-adjusted availability data. The relative composition across food groups is the same between the two datasets.

Our method of estimating waste fractions is detailed in the methods section and in a dedicated section in the supplementary information entitled “SI1. Adjusting food availability statistics for food waste”. We comment on this procedure in more detail in response to your comment 3-11 below, and following this and your comment 3-14, we uploaded our country-level estimates of food intake (with and without waste) to a public repository.

Comment 3-6:
On top of this, I would like to see a reflection on the methodology you used to assess the amount of nutrients embedded in processed foods, especially providing the conversion factors used.

Reply to Comment 3-6:
The processing fractions for red meat into processed and unprocessed red meat, and those for grains into whole and refined grains were derived from the GDD at a weight bases (grams) and applied to the estimates of intake in both grams and calories. The implicit assumption is that the calorie densities between the converted and conversion products are similar, which is justified for grains and red meat in absence of regionally comparable data that would indicate otherwise (e.g., the GDD only contains estimates in grams). For dairy, the calorie densities between conversion product (milk) and converted products (cheese, yoghurt) are very different, which is why we derived conversion ratios at a calorie basis, then split products at a calorie basis, and finally calculated the corresponding weights (in grams) by using calorie densities of the specific products (i.e., of cheese and yoghurt), with both the processing ratios and the calorie densities derived from the supply utilisation accounts. We described this in the methods section as follows and indicated the bases (kcal or grams) in Table S7:

> "The normalised estimates of food intake describe intake in terms of primary commodity equivalents (e.g., all milk needed to produce the consumed levels of dairy products). Where available, we used processing ratios to supplement the estimates of primary commodities with those of processed foods. We used FAO's supply utilisation accounts to derive processing ratios that describe the proportion of milk processed into dairy products (cheese and yoghurt)[17], and we used GDD estimates from dietary surveys to derive processing ratios for partitioning all grains into refined and whole grains, and for total red meat into processed and unprocessed red meat[32]. Table S7 in section SI3 provides an overview of the processing ratios used."

Following your comment, we clarified at what basis these conversion ratios were applied by noting:

> "Because the calorie densities of dairy products differ, we split those according to calories from each products and converted them to the equivalent weights using product-specific calorie densities adapted from the FAO[17]. For grains and red meat, we applied the same processing ratio to weights and calories."

We edited the supplementary information (section SI3) accordingly. For dairy processing, we added:

> "We used the calorie densities of each product (also adopted from the Supply Utilisation Accounts) to convert from calories to the equivalent weights in grams."

For red-meat processing, we added:

> "In absence of regionally comparable calorie densities of refined grains and processed meat (the GDD provides estimates in grams only), we used the same processing ratios for grams and calories, implicitly assuming equal calorie densities between products."

Comment 3-7:
Line 232-235: As the survey has a more limited coverage of food groups may you further explain how this procedure was performed? Additionally, how does this relate or cope with the differences/inconsistencies in the surveys estimates remarked in the introduction and results section?

Reply to Comment 3-7:
Sure, we explained how we mapped the food groups in the methods section which we revised for clarity. The relevant section reads:

> "We then calculated ratios of food intake across the socio-demographic groups and for the food groups covered by the GDD (Table S8) and mapped those ratios to the food groups covered in our database (Table S9)."

The table (Table S9) lists to what specific food groups the estimates from the GDD were applied. For example, the sociodemographic trends for grains (GDD food group) were

applied to all grain commodities (wheat, rice, maize, other grains) represented in the processed FBS data.

The caveats related to dietary surveys in the GDD apply also to the socio-demographic ratios derived from them. However, we aimed to make them more biophysically plausible by energy-correcting them in the same way that we corrected the population-averaged intake at the country level. Specifically, we normalised the GDD estimates of intake for the different population groups by the independent estimates of total energy intake which cover the same population groups. In addition, we energy-corrected the final estimates for the different socio-demographic groups one more time after the mapping of socio-demographic trends to account for differences in food group coverage between the two sources. We added a dedicated explanation in the supplementary information on this clarified our procedure in revised methods section as follows:

> "To align the GDD estimates with biophysically grounded estimates of energy intake, we used the estimates of energy intake that are in line with current biophysical requirements per population group in each country[39] to re-normalise the GDD estimates to those levels (Tables S5-S6). [..] To ensure that the mapping between food groups and source data preserved the overall levels of energy intake, we also normalised the final estimates for each socio-demographic group to their estimated energy intakes. Section SI4 provides additional descriptions and an overview of the data used in these steps."

We acknowledged the remaining caveats in the discussion section as follows:

> "[O]ur new estimates of food intake are also subject to several caveats. Many of them carry over from those associated with the data sources used in their construction, including estimates of food availability, food waste, estimated energy intake, and dietary surveys."

Comment 3-8:
Line 261-264: An additional inconsistency of the survey-based estimates was that estimates for specific food groups such as red meat exceeded food-availability statistics (with waste included) for upper middle-income countries where statistics on food availability are relatively good.
How do the authors deal with this potential inconsistency?

Reply to Comment 3-8:
In the lines you refer to, we explain why we did not feel comfortable using the GDD estimates for informing absolute values of intake. We clarify this at the start and end of the paragraph as follows:

> "An alternative to deriving food intake from statistics on food availability could have been to adopt estimated intake from dietary surveys, at least for the foods reported in global databases such as the GDD. However, [list of inconsistencies]. Based on these consistency checks, we decided against using dietary surveys for deriving a complete diet proxy, and instead used their variation across sociodemographic groups to supplement our main estimates."

Comment 3-9:
Lines 309-312: Although our method of deriving intake can also be used to derive changes in overall wastage rates over time (by subtracting energy intake from calorie availability), it cannot inform commodity-specific changes.
I do not comprehend why you state that you cannot address commodity-specific changes of waste rates over time. In principle you have gross intakes by commodity (FBS) and commodity-specific waste rates (Gustavsson et al) so why couldn't you calculate this?

Reply to Comment 3-9:
We used commodity-specific wastage rates derived by Gustavsson and colleagues around the year 2010. Those are percentage rates that apply to different food commodities. We applied those to the FAO's food availability estimates for all years in our time series and then energy-corrected the overall intake for each population group based on the external estimates of energy intake that are in line with measured levels of body weight, height, and physical activity. The energy-correction is done at the overall food level, meaning it applies to all foods in proportion. This can inform how all rates of food waste changed, but not how particular ones did. Following your comment, we clarified this in the sentence you referred to as follows:

> "Although our method of deriving intake can also be used to derive changes in overall wastage rates over time (by subtracting energy intake from calorie availability), it cannot inform commodity-specific changes apart from those overall shifts."

Please also see our methodological clarifications in response to your comment 3-11 below.

Comment 3-10:
Lines 312-216: New estimates of food waste have recently been compiled, but not yet standardized in the same way as the FAO's, which prevented us from using them. For example, they used a different split across the food chain, and their overall estimates resulted in levels of food consumption inconsistent with observed trends in body weight across regions.

The used food waste fractions from FAO (Gustavsson et al., 2011) have been widely criticized and addressed as inconsistent by FAO itself (FAO, 2019). As stated by the authors, more recent waste fractions are available from the literature (e.g. Gatto and Chepeliev, 2024). Such estimates provide the same framework of Gustavsson (share of lost or wasted food by region, food type, and supply chain stage), overcoming however the much discussed limitations of Gustavsson et al, (i.e. non-food use of food product considered as waste and FLW shares computed only on edible part of food commodities). The split across the supply chain adopted by Gatto and Chepeliev is the same of Gustavsson et al. Net intake levels derived by applying Gatto and Chepeliev's food waste shares remain within the plausible range of daily calorie intakes (as illustrated in the SI of their publication). For this, as this estimates are a collection of waste fractions derived from more recent studies, I encourage the authors to test the application of this more up-to-date waste fractions, with the aim of improving the accounting of food waste across countries.

Reply to Comment 3-10:

Thank you for the comment. We assessed in some detail whether the new waste estimates were usable in their current form. However, two main issues made us decide against that. First, the waste estimates provided by Gatto and Chepeliev parse the different steps along the food/waste chain differently and arrive at a substantially different split between the ratio of foods that is lost before it reaches the market and the ratio that is wasted after it reaches the market. Their fractions also apply exclusively to the foods produced in a country. Both aspects make them not applicable to estimates of foods available in a country which already have food loss subtracted and food trade accounted for (as FAO's food balances have).

Conversely, using the estimates by Gatto and Chepeliev would require dropping the FAO's estimates of food loss and using an agricultural trade model. FAO's estimates of food loss are provided annually as part of their food balances which we used as a primary input to our estimates. We therefore did not want to drop those estimates. We also wanted to minimise additional assumption that come with the use of an external agricultural trade model. For example, the GTAP model's input-output database used by Gatto and Chepeliev is based on economic accounts which have shortcomings when deriving actual quantities from them and are also less recent than the data provided by the FAO.

Second, the estimates of final food demand provided by Gatto and Chepeliev are not in line with measured trends in body weight and BMI across regions, which made us doubt their consistency. For example (and there are many more), they reported estimates of net food supply in 2014 that were larger for many East Asian countries (e.g., Thailand, Malaysia, Cambodia) than for the USA, in contrast to the USA having a much larger prevalence of overweight and obesity and, related to that, much larger energy requirements (also after accounting for differences in physical activity). We communicated these inconsistencies (which curiously are not present in the older Gustavsson estimates) to the authors, and they agreed with us that they present a problem. We still think the study by Gatto and Chepeliev presents a much-needed initiative for updating waste fractions, so we did not want to overly criticise their study in our resource paper. Instead, we hope to be able to use updated estimates in the future and therefore decided to focus on that in our discussion.

Comment 3-11:
METHODOLOGY
A general remark regards the application of waste fractions. It is unclear if the authors apply a total waste fraction to overall intakes by country or if this procedure is done at the commodity level. In principle, given the amount of information from the FBS, Gustavsson et al., you should be able to apply commodity-specific waste fractions to commodity-specific intakes. If you don't do so, please explain why. Generally, please add a more detailed description of this procedure. If a total waste fraction is adopted, please describe the method used to calculate the average waste fraction across commodities.

Reply to Comment 3-11:
Sorry this was a bit unclear. We first used the commodity and region-specific waste fractions provided by Gustavsson and colleagues to estimate food waste for each food commodity and country. We then normalised the overall level of food intake to the external estimates of total energy intake, which introduced an overall scaling parameter. The scaling was fairly minimal for 2010, the year waste fractions were derived initially, but it moderately increased for later years, indicating greater wastage rates (see Table S6). We explained this in the

methods section as follows, now clarifying that the first calculation of food waste was conducted at the commodity level:

> "The FBS data are adjusted for estimated losses during agricultural processing, storage and transportation, but not for food waste occurring at the level of retailers and households. We used the waste-accounting methodology and parameters developed by the FAO to estimate for each commodity and country the amount of food wasted during distribution and consumption[49], and subtracted those from the estimates of food availability. Section SI1 and Tables S2-S3 in the Supplementary Information provide a detailed description of the method and parameters used.
>
> This method of deriving a proxy for dietary intake starts at the level of reported values of foods produced, traded, and processed, and subtracts relevant portions across the food chain on the way to consumption, all of which introduce unquantifiable levels of uncertainties. To ensure estimated intake is biologically plausible, we introduced a further step in our analysis at the level of food consumption. In particular, we normalised food intake to estimates of total energy intake[39]. The later was based on calculating the energy intake required to sustain measured levels of body weight, height, and physical activity. For the normalisation, we adjusted estimated food intake by the overall calorie ratio between waste-adjusted availability estimates and the estimates of total energy intake (Tables S5-S6). Section SI2 provides a detailed description of the estimates of total energy intake."

Comment 3-12:
Lines 347-349: How does this step deal/account for the inconsistency of dietary surveys across specific commodities outlined across the paper?
Please illustrate (in the SI) how you calculate how much is consumed directly and how much through all processed foods that are not reported in the SUA? Like Infant food or miscellaneous. In this, please list all processed foods accounted in this calculations.
Lines 391-392: How about other processed foods? How are they treated?

Reply to Comment 3-12:
The first lines you are referring to provide a summary of the methods that are then detailed further below. As explained in our responses and in the main text, we deal with the main uncertainty related to dietary surveys by not using their absolute values for our estimates and by normalising their estimates to external estimates of energy intake before deriving from them the variations in intake across socio-demographic groups that we then use for our estimates. We explain this in the introduction, methods and discussion sections. In the summary of methods you referred to, we note:

> "Third, we derived socio-demographic trends in intake from dietary surveys normalised to estimates of total energy intake by population group."

Further below in the methods, we provide additional details on why we conduct the energy-normalisation (please see the section quoted in response to your previous comment).

When it comes to processed foods, we only account for processed grains, red meat, and dairy. The reason for this is that we were able to derive regionally comparable conversion

ratios for those foods and they are regarded as important in some dietary assessments, for example in health and nutrition-related assessments. We offer them in addition to the estimates provided in primary commodity equivalents. We did not aim to translate all foods into processed foods, so any other food group is not converted and used as reported in the food balance sheets, before being aggregated and waste and energy-corrected. We explain the coverage of processed foods in the methods section as follows:

> "The normalised estimates of food intake describe intake in terms of primary commodity equivalents (e.g., all milk needed to produce the consumed levels of dairy products). Where available, we used processing ratios to supplement the estimates of primary commodities with those of processed foods. We used FAO's supply utilisation accounts to derive processing ratios that describe the proportion of milk processed into dairy products (cheese and yoghurt)[17], and we used GDD estimates from dietary surveys to derive processing ratios for partitioning all grains into refined and whole grains, and for total red meat into processed and unprocessed red meat[32]."

We detailed the aggregation of the commodities listed in the food balance sheet (including infant food and miscellaneous) in the supplementary information (section SI1 and in particular Table S1), which we refer to at the start of the detailed methods description:

> "For further processing, we aggregated the commodity detail to a list of 37 food groups (Table S1)."

Comment 3-13:
Line 432: Please use either "To illustrate" or "For illustrating" .

Reply to Comment 3-13:
Thank you for catching that. We corrected the typo to "For illustrating".

Comment 3-14:
SUPPLEMENTARY INFORMATION
Please add an overview of country-specific food waste levels calculated in this process. Please list the waste fractions used by food product and the food intake estimates before and after the food waste fractions are applied.

Reply to Comment 3-14:
The supplementary information is used to provide overviews of data as otherwise it would become too large. For example, the country and commodity-specific estimates of food waste you requested would run over 37 (number of food groups) x 171 (number of countries) = 6,327 lines. For the waste fractions, an overview by commodity, stage in the food supply chain, and region is provided in Table S3, together with a detailed description of how they are applied in section SI1.

As noted in the manuscript, we will make the full estimates of food intake available upon publication. For the meantime, we uploaded them onto an online folder: https://drive.google.com/drive/folders/1XF2btDT3sPyuz9Iv3pXinvuGtIkCDWcP?usp=sharing .

Following your comment, we momentarily added to this the estimates of food availability/demand based on FAO data which conceptually include waste. Normally, one can calculate total waste fractions from dividing demand by intake. However, because the energy-correction of waste-adjusted availability data increased intake in low-income countries (Table S6), the intake estimates can in some situations become larger than the estimates of demand that are reported in FAO's food balance sheets. This makes sense because the demand statistics do not account for food intake from subsistence production. In contrast, our new estimates account for subsistence production (proportionally assigned to all foods in our main proxy, and selective assigned to vegetables and fish in one of the additional proxies). Because of this, we do not think that deriving waste fractions in this way is very meaningful and would rather work with updated direct estimates once they become available (see, e.g., our reply to your comment 3-10). We would also like to note that our aim in this study was to derive biophysically plausible estimates of food intake, and not new estimates of food waste.

Comment 3-15:
Table S3. Where do this percentages of processed foods in overall food consumption come from?

Reply to Comment 3-15:
All data in that table come from the report (including its technical annex) on food waste by Gustavsson and colleagues as referenced in the text (ref 2).

Response to Reviewer #1

Comment 1-0:
Thanks to the authors for addressing the feedback, making revisions, adding details to the manuscript, and/or clarifying our questions. Some additional comments for consideration:

Reply to Comment 1-0:
Thanks a lot for another round of feedback which we think helped to further improve the manuscript. Please find our point-by-point response below.

Comment 1-1:
- "The most commonly used data in impact assessments are estimates of how much food is available in a country": this may well be true, but just wanted to check - are these quantifiably the most commonly used, or just commonly used?

Reply to Comment 1-1:
Good point. When it comes to impacts assessments, that's certainly the case, but we don't know of any quantification of this. Following your comment, we therefore changed the statement to:

> "One of the most commonly used [..]."

Comment 1-2:
- I still think the terminology referring to the various datasets and underlying resources is unclear. There are close to 150 instances of 'estimates' or 'the estimates' in the paper. The authors replied that they "provide a description linking these terms to their abbreviations in the first results section", though that section does not provide that - there is no standard set of definitions and abbreviations. The paper still uses a variety of terms to refer to a single dataset.

For example, references to the GDD include: survey estimates, estimates based on dietary surveys, survey-based data, global estimates of food intake, estimated intake from dietary surveys, intake from surveys

References to FAO FBS include: Food availability statistics, availability-based, food availability data, estimates of food availability, national estimates, availability estimates, food balances, estimates of food balances, FBS-based estimates.

Please consider using simplified and consistent terms like GDD, GDD-IA and FBS. This will reduce word count, clarify which dataset is being referred to (e.g. instances of 'national estimates' that I think refer to FBS data, but this could refer to any of the various data that are at national level). It is also helpful as it differentiates when authors refer to survey/data types generally (when they discuss biases or strengths of a certain data type or survey format) versus when they are referring to the specific GDD/FAO databases. Again, all of this would provide a smoother, more coherent reading experience.

Reply to Comment 1-2:
Thank you for your comment and suggestion to streamline the presentation of results. Our

preference was to use explanatory language instead of abbreviations when describing the different data sources to have a reading experience accessible to a general reader of Nature Food. However, we do agree that this can sometimes be prone to misunderstanding. Following your comment, we now included the abbreviations of the dataset used at first mention in each paragraph in the relevant result sections, and we clarified which national estimates we were referring to be more specific. We hope this is a good compromise and clarifies the intended meaning. The following provides a few examples of the changes made:

> "At the global level, the uncertainty intervals of the new GDD-IA estimates of food intake overlapped with the uncertainty intervals of dietary-survey estimates from the GDD for most food groups"

> "We conducted a series of impact assessments (see *Methods*) to illustrate the relative differences between the new database (GDD-IA), the global database of dietary surveys (GDD), and waste-adjusted food availability data (FBS). In a health assessment based on a comparative risk assessment of dietary risks (Figure 5a, Table S13), we found that using the GDD-IA resulted in a lower estimate of deaths attributable to dietary risks as when using the GDD data (-750,000 globally; -12%), but in a higher number as when using FBS-based data (+780,000; +17%)."

> "In an environmental assessment based on life-cycle assessments of environmental footprints, the estimated impacts were similar between the GDD-IA and FBS-based data, but there were large differences between the GDD-IA and survey-based GDD estimates (Figure 5b, Table S14)."

> "For the former, we mapped the variation in intake across sex, age groups, and urban/rural residence inferred from the GDD dietary surveys[32] to the food-group detail of the FBS-based national estimates"

Our convention is that we use "-based" to indicate that some processing has been conducted such as the harmonisation of dietary surveys in the GDD or the waste-adjustment that we applied to the FBS data.

Comment 1-3:
- Please consider differentiating and making more clear, what is an existing data resource versus a data manipulation/adjustment process that authors conducted. Examples:

"Estimated intake in the GDD was normalised to a constant level of energy intake" [I assume this already the characteristic of that dataset, rather than authors conducting this as a step. Simply changing to "is normalised", or similar, would help clarify]

"The LCAs were standardised by harmonising system boundaries (from inputs to retail) and gap-filling missing steps along the supply chain, which made use of auxiliary estimates (e.g., of post farm processes) and dedicated process-based models (e.g., of nitrate leaching). FAO data on food production and yields were used to scale to official statistics and regionalise the estimates, and FAO data on trade were used to derive consumption-based footprints by 16 538 commodity and region" [In the reviewer replies, the authors explained that they are just describing the Poore/Nemececk data ... but the way it is worded is indistinguishable from the

many other data adjustment steps the authors describe they conducted]

Reply to Comment 1-3:
Thank you for the comment. We tried to use active voice when describing what we did and passive voice when something was done by others. We agree this can be further clarified by using different tenses. Your suggestion of using present tense works very well in the first instance, but we think using present perfect works a bit better for the second instance. For consistency, we therefore adopted this tense for all cases where we report on steps done by others. We hope this clarifies the intended meaning. The two instances you mentioned above now read as follows:

> “Estimated intake in the GDD has been normalised to a constant level of energy intake”
>
> "The LCAs have been standardised by harmonising system boundaries (from inputs to retail) and gap-filling missing steps along the supply chain, which made use of auxiliary estimates (e.g., of post farm processes) and dedicated process-based models (e.g., of nitrate leaching). FAO data on food production and yields have been used to scale to official statistics and regionalise the estimates, and FAO data on trade were used to derive consumption-based footprints by 16 538 commodity and region”

In one instance when describing how we constructed additional proxies, we changed passive voice to active voice to signal that we conducted the calculations:

> “We constructed the subsistence-adjusted proxy as follows. First, we adapted the intake values for the targeted foods in the specific regions from the survey-based proxy. Second, we adapted the intake values for the remaining foods and regions from the main proxy. Third, we calculated overall energy intake and normalised it to levels in line with biophysical requirements for consistency.”

Comment 1-4:
- This specific example here may be a combination of the considerations I have above:

“We disaggregated national food intake by age group, sex, and urban/rural residence based on trends in intake inferred from dietary surveys and normalised to estimated energy intake per population group. We sourced the differences in dietary intake by sociodemographic group from the GDD.”

Reading this, I'm confused on what step is being described in the data adjustment (apart from the energy intake normalisation). The GDD already has intake disagregated by age group, sex, urban/rural residence. Does "national food intake" refer to the starting database (if so, it would be simpler to say GDD or FBS), or does it just refer generally to data at a national level. The phrase "intake inferred from dietary surveys" is already shorthand that the authors used the GDD (so would be simpler to say this), and would not need to repeat the same point again in the next sentence "We sourced the differences in dietary intake by sociodemographic group from the GDD." This is a small example, but many of these around

the paper.

Reply to Comment 1-4:
Thank you for highlighting this statement. These sentences are from the introduction of the section “Estimating socio-demographic variation in food intake” in the methods, following the section titled “Estimating national-level food intake”. We thought this makes clear that the estimates we refer to are those described in the previous section. Following your comment, we now start the first sentence with:

> “We disaggregated the estimates of national food intake derived above [..].”

The rest of the sentence provides a general explanation on what was done as an introductory sentence which are usually kept general, which is followed up with the specifics, including the relevant abbreviations. The body of the paragraph describes the GDD data, which has not been introduced before in the methods section, and the next paragraph explains the specific steps we followed for processing and using the GDD data:

> “We processed the GDD data in several ways to be able to consistently combine them with our national-level estimates of food intake. [..]”

Coming back to the highlighted sentences, you are right that the GDD data are already differentiated by sociodemographic group. We derived trends in intake from those data (a simple calculation of proportional changes, but still an active step in our calculations), and we then used those changes to disaggregate the waste-adjusted FBS data, and finally normalised those to the EER data on total energy intake. We describe these steps in the second paragraph mentioned above, for example:

> “We then calculated ratios of food intake across the socio-demographic groups for the food groups covered by the GDD (Table S8) and mapped those ratios to the food groups covered in our database (Table S9).”

To be consistent with what we did and what the source data was, we changed the sentences you highlighted to:

> “We disaggregated the estimates of national food intake derived above by age group, sex, and urban/rural residence based on trends in intake we inferred from survey-based estimates and normalised to estimated energy intake per population group. We sourced the survey-based estimates of dietary intake by sociodemographic group from the GDD[32]. The GDD contains [..].”

We hope the description is clearer and more consistent now.

We also went through the whole manuscript again and clarified the steps in our calculations and the multiple comparisons by using a consistent terminology that we described in the responses to your previous comments. This included supplementing descriptions of data by their abbreviation, being consistent what estimates were based on what source, and more clearly differentiating our own steps by using active voice and describing those done by others by using passive voice and present perfect as the tense.

Comment 1-5:
I hope these are constructive, and would help with reproducibility of the impressive analysis by others.

Reply to Comment 1-5:
Thanks a lot for your constructive feedback. We do appreciate the detailed and specific comments and suggestions you provided and think they did help in clarifying the manuscript.
